# *De Novo* Assembly of Expressed Transcripts and Global Transcriptomic Analysis from Seedlings of the Paper Mulberry (*Broussonetia kazinoki* x *Broussonetia papyifera*)

**DOI:** 10.1371/journal.pone.0097487

**Published:** 2014-05-21

**Authors:** Peng Xianjun, Teng Linhong, Wang Xiaoman, Wang Yucheng, Shen Shihua

**Affiliations:** 1 Key Laboratory of Plant Resources, Institute of Botany, the Chinese Academy of Sciences, Beijing, PR China; 2 University of the Chinese Academy of Sciences, Beijing, PR China; Cankiri Karatekin University, Turkey

## Abstract

The paper mulberry is one of the multifunctional tree species in agroforestry systems and is also commonly utilized in traditional medicine in China and other Asian countries. However, little is known about its molecular genetics, which hinders research on and exploitation of this valuable resource. To discern the correlation between gene expression and the essential properties of the paper mulberry, we performed a transcriptomics analysis, assembling a total of 37,725 unigenes from 54,638,676 reads generated by RNA-seq. Among these, 22,692 unigenes showed greater than 60% similarity with genes from other species. The lengths of 13,566 annotated unigenes were longer than 1,000 bp. Functional clustering analysis with COG (Cluster of Orthologous Groups) revealed that 17,184 unigenes are primarily involved in transcription, translation, signal transduction, carbohydrate metabolism, secondary metabolism, and energy metabolism. GO (Gene Ontology) annotation suggests enrichment of genes encoding antioxidant activity, transporter activity, biosynthesis, metabolism and stress response, with a total of 30,659 unigenes falling in these categories. KEGG (Kyoto Encyclopedia of Genes and Genomes) metabolic pathway analysis showed that 7,199 unigenes are associated with 119 metabolic pathways. In addition to the basic metabolism, these genes are enriched for plant pathogen interaction, flavonoid metabolism and other secondary metabolic processes. Furthermore, differences in the transcriptomes of leaf, stem and root tissues were analyzed and 7,233 specifically expressed unigenes were identified. This global expression analysis provided novel insights about the molecular mechanisms of the biosynthesis of flavonoid, lignin and cellulose, as well as on the response to biotic and abiotic stresses including the remediation of contaminated soil by the paper mulberry.

## Introduction

The paper mulberry is a member of the Moraceae family and is naturally distributed in Eastern Asia and pacific countries. The trees are dioecious and deciduous and serum-secretive. As a pioneer species, the paper mulberry has a great adaptability to climates and soils and strong resistance to pests and diseases, among other characteristics. It is the ideal tree species for gardening and ecological reclamation, and widely used in papermaking, livestock, medicine and other industries.

The paper mulberry is a shallow-rooted tree with advanced lateral roots but no obvious taproot. Due to its fast growth and strong adaptability, the paper mulberry is commonly used for the ecological afforestation and landscaping along highways, in mined areas and on barren land [1]. The tremendous endogenous distribution range of this tree shows its ability to thrive in various climates. The paper mulberry is even considered an invasive plant because it has quickly spread throughout areas of Florida [2] and Ghana [3].

The bark of the paper mulberry is often used to make paper and envelopes in the handicraft industry. The average length of a phloem fiber is 7.45 mm, and that of xylem is 0.58 mm, which makes it an excellent material for the production of high-quality paper [4]. In addition, bark from the paper mulberry has already been grown as a cash crop, providing a successful example of domestication of a forest product [5]. Indeed, the rotation of upland rice and paper mulberries, from which the inner bark is harvested, increases the productivity of a traditional slash-and-burn system [6]. Importantly, paper mulberry may be an alternative crop intercropped with pineapple, sugar cane and kenaf in agroforestry system [7].

Because of its abundance in proteins, the leaves of the paper mulberry have been used as a kind of non-conventional forage, which is superior to compound feed and reduces the cost of feeding livestock and poultry [8]. In addition, the fruit extract can also be used in dietary supplement preparations, as a food additive, or to prevent oxidation in food products [9].

Due to the high contents of flavonoids and other secondary products, the paper mulberry has long been used in Chinese traditional medicine for the treatment of inflammatory disorders, especially for systemic septic inflammation, as well as chronic bronchitis [10]. For example, broussochalcone, one of the medicinal compounds of the paper mulberry, is a powerful antioxidant with versatile free radical-scavenging activity, and is able to directly scavenge superoxide anions and hydroxyl radicals [11]. Papyriflavonol-A, also found in paper mulberries, has an effective antifungal activity and strong antibacterial activity and is used in traditional Asian medicine to treat microbial infections. This notion suggests a strong potential for prenylated flavonoids as antimicrobial agents as well as anti-inflammatory agents [12]. Moreover, ethanol extracts of paper mulberry could significantly interfere with the development of *Plutella xylostella* population, and has the potential to be an effective means to control this insect pest, which attacks cruciferous crop vegetables, including broccoli, cabbage, and turnips [13].

Enhanced photosynthetic performance is observed during re-watering periods, which allows for a complete recovery of paper mulberries from successive intermittent drought stresses. The paper mulberry can effectively reduce the osmotic stress of hypersaline soil by absorbing and accumulating NaCl [14]. In addition, the contents of carbohydrates, amino acids and phytohormones in the leaves of paper mulberry are affected by traffic pollution, suggesting that paper mulberries can physiologically regulate their metabolism to adapt to or resist traffic stress [15]. As a landscape plant, the paper mulberry has been grown broadly in polluted areas due to its relatively high phytoremediation capacity to clean up strontium and other toxic metals from contaminated soils [16]. The surface properties of the leaf powder of paper mulberry is suitable for the metal adsorption process, which makes it as an ideal adsorbent to remove heavy metal ions from aqueous solutions [17].

In order to facilitate the collection and evaluation of germplasm resources, a male-specific AFLP (Amplified fragment length polymorphism) marker has been developed and can be used for sex identification in paper mulberry [18]. Genetic diversity revealed by SRAP (Sequence related amplified polymorphism) markers and cluster analysis have shown that there is a relationship between the genetic variation and geographical distribution [19]. These results provide a reference for mapping of QTL (Quantitative Trait Locus) regions and breeding of paper mulberry.

However, our knowledge about the genetic basis of the beneficial traits show by the paper mulberry is still lagging behind advances in understanding its physiology and biochemistry. The biosynthesis pathways of flavonols and other medical components are not fully elucidated. The molecular mechanism underlying its strong adaptability and tolerance to biotic or abiotic stress remains poorly understood, which presents a challenge to the further exploitation of the paper mulberry for human needs. This is mostly caused by the fact that few genetic studies have been done on this species, due to its long lifecycle [20]. In recent years, the development of Next Generation Sequencing (NGS) has provided a means by which we can rapidly generate significant amounts of genetic data. The Illumina platform is able to produce millions of reads in one run and provide reads from 100 to 250 bp in length with lower cost. This technology is suitable for de novo sequencing [21], [22], re-sequencing [23], transcriptomics [24] and metagenomic sequencing [25]. In 2011, the Trinity program for transcriptome reconstruction has been developed to assemble a transcriptome from NGS data when no reference genome is available [26]. De novo assembly of RNA-seq data enables researchers to study the transcriptome without a genome as reference [27], which has been successfully applied in screening and identification of secondary metabolite biosynthesis [28], differential tissue expression [29], stress response [30] and agronomic traits related genes [22], [31].

In this paper, we adopted a mixed sampling strategy to obtain the transcriptional profile of three vegetative tissues of the paper mulberry. About 54,638,676 Illumina reads were de novo assembled for the first time to build the transcriptome of the paper mulberry. Sequence information for those genes involved in the biosynthesis of flavonoids, cellulose and other compound can be used for metabolic engineering of the paper mulberry. The large number of transcripts reported in the current study additionally serves as an invaluable genetic resource for crop improvement of paper mulberry as well as other plants.

## Materials and Methods

### Plant Material and RNA Extraction

Plantlets of the paper mulberry, regenerated from the young leaves, were cultured on MS culture media (Murashige and Skoog) at 26°C and a 14/10 h photoperiod (day/night). Leaves, stems and roots were collected from 3-month-old plantlets. A mixed sampling strategy was adopted to eliminate differences between individuals. Total RNAs were isolated with TRIzol® Reagent (Life technologies, Shanghai, China) from each sample according to the manufacturer’s instructions. RNAs were treated with RNase-free DNase I (Takara, Dalian, China) to remove the DNA residues. The quality and purity of RNAs were assessed with OD260/230 ratio and RNA integrity number (RIN) by using the NanoDrop 2000 (Thermo Fisher, Waltham, USA) and the Agilent 2100 Bioanalyzer (Agilent Technologies, Santa Clara, USA), respectively.

### cDNA Library Preparation and Sequencing (RNA-seq)

mRNAs were purified from the mixed high quality total RNA (3 µg) using Oligo (dT) RNA purification. Beads and impurities were removed from the hybridized sample with a series of washes in low-salt solution. The purified poly (A) RNAs were then dissolved into a Tris-based buffer, precipitated with 70% ethanol and re-suspended in buffer. First strand cDNAs were synthesized using Oligo (dT) primer, then second strand cDNAs were synthesized using RNase H and DNA polymerase I. Double stranded cDNAs were random fragmented using Nebulizer, then repaired and added an adenine base to the 3′ end. Two different adapters were ligated to the cDNA fragments, which were then purified using second strand AMPure XP beads (Beckman Coulter, California, USA). cDNA templates were enriched by multiplexing PCR with primers 1.0 and 2.0. Barcodes were added to each library during amplification.

cDNA libraries were validated using a high sensitivity chip on the Agilent 2100 Bioanalyzer (Agilent Technologies, Santa Clara, USA). The cDNA library was quantified using the PicoGreen Assay and qPCR. The libraries were clustered on a flow cell using the cBOT, an Illumina automated clonal cluster generator. After clustering, the samples were loaded on the Illumina Genome Analyzer IIX platform and sequenced. All three cDNA libraries were run on one lane. The samples were sequenced using a paired-end reads with 201 cycles. Initial base calling and quality filtering of the Illumina GA image data were performed using the default parameters of the Illumina GA Pipeline GERALD stage (http://www.illumina.com). Additional filtering for reads, including homopolymers and low quality bases, was performed using custom-written code. Raw sequence data were generated by the Illumina pipeline and are available in NCBI’s Short Read Archive (SRA) database (http://www.ncbi.nlm.nih.gov/Traces/sra/sra.cgi) under accession number SRA039814.

### De novo Transcriptome Assembly

The pipeline of the transcriptome experimental and bioinformatics analysis is shown in Fig. S1. All the following analyses were based on the clean reads. Transcriptome de novo assembly was carried out with Trinity [26]. Based on a pilot assembly, the optimal parameters were identified as k-mer of 25 and group pairs distance of 500. This generated the optimum transcript contiguity while preserving transcript diversity. Using these parameters, the Illumina reads generated from the 3 cDNA libraries were de novo assembled into contigs by Trinity. Next, the contigs were connected using N to represent unknown sequences between each two contigs to generate scaffolds. Paired-end reads were used again for the gap filling of scaffolds to get sequences that had least Ns and could not be extended on either end. Such sequences were defined as transcripts. Based on the Trinity assembly results, a longest transcript was selected from the potential alternative splicing transcripts as the unigene sequence of the sample. Blat (The BLAST-Like Alignment Tool) was applied to make alignments among three samples. The longest unigene of these 3 samples was considered as the unigene of paper mulberry. Finally, all of the clean reads were pooled together and assembled to form the global transcriptome of the paper mulberry.

### Gene Expression Analysis, Annotation and Functional Classification

For gene expression analysis, the expression level of each unigene in each sample was calculated by quantifying the number of Illumina reads that mapped to transcriptome of paper mulberry with default parameters. The raw gene expression counts were normalized using the RPKM method (Reads per kb per million reads) [32].

In the functional annotation, unigenes were first aligned by Blastx to protein databases nr, Swiss-Prot, TrEMBL, KEGG and COG (using E≤1e-5 as the threshold), retrieving proteins with the highest sequence similarity with the given unigenes along with their protein functional annotations. In addition, Blast2GO program (E≤1e-5) was used to get GO annotation of unigenes. After GO annotation for every unigene, we used WEGO software to do GO functional classification for all unigenes and to understand the distribution of gene functions of the species from the macro level. The URL of these databases was as follows: http://www.geneontology.org/, http://www.genome.jp/kegg/, http://www.ncbi.nlm.nih.gov/COG/, http://www.uniprot.org/ and http://www.ncbi.nlm.nih.gov/.

### Identification of Differentially Expressed Genes

For screening of differentially expressed genes, p values corresponding to differentially expressed genes (DEGs) were obtained via a general Chi squared test that was performed by using IDEG6 (http://telethon.bio.unipd.it/bioinfo/IDEG6/). The threshold of p value in multiple tests was checked through manipulating the false discovery rate (FDR) value. The unigenes with ratios of RPKM between samples greater than 2 were considered to have significant differences in expression.

In gene expression profiling analysis, GO enrichment analysis of functional significance was applied for hypergeometric test to map all DEGs to terms in GO database, in order to determine significantly enriched GO terms in DEGs compared to the transcriptome background of the paper mulberry. The formula use was: 
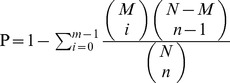
 where N denotes the number of all genes with GO annotation; n, the number of DEGs in N; M, the number of all genes that are annotated to the certain GO term; m, the number of DEGs in M. KEGG, a large pathway-related database, was used to identify significantly enriched metabolic or signal transduction pathways in DEGs compared with the whole transcriptome background. The formula used was the same as for GO analysis. Here N is the number of all genes with KEGG annotation; n, the number of DEGs in N; M, was the number of all genes annotated to the specific pathway; and m, the number of DEGs in M. The p value of a test measured the value of FDR when the particular test was called significant (http://genomics.princeton.edu/storeylab/qvalue/).

### Identification of Simple Sequence Repeats (SSRs)

The MISA (MIcroSAtellite identification tool, http://pgrc.ipk-gatersleben.de/misa/) program was used to identify SSRs in the transcriptome of the paper mulberry. Seven types of SSRs (mono-nucleotide, di-nucleotide, tri-nucleotide, tetra-nucleotide, penta-nucleotide, hexa-nucleotide and compound) were screened by MISA from the unigenes. The parameters of MISA in this analysis were set as follows: 1/10, 2/6, 3/5, 4/5, 5/5 and 6/5 (unit size/minimize repeats). For the compound SSRs, the interruption was defined as 100; namely, the distance of between two SSRs was not longer than 100 base pairs.

### Validation by qPCR

Real time PCR (qPCR) was used to verify the assembly and the DEG results of the RNA-seq. Twelve unigenes were randomly chosen for verification (Table S1). RNA used for validation was the same as that for RNA-seq. The first-strand cDNA for each sample was prepared from 1 µg of total RNA using SuperScript II reverse transcriptase (Takara, Dalian, China) following the manufacturer’s instructions, and template cDNAs were diluted 5-fold for PCR. Gene-specific primers were designed using the Primer5 program and are listed in Table S2. Samples and standards were run in triplicate on each plate using the SYBR® Premix Ex Taq™ II kit (Takara) on a MX3000P Real-Time PCR System (Agilent Technologies, Santa Clara, USA) following the manufacturer’s recommendations. Real-Time PCR was performed in a 20 µL reaction volume containing 6.8 µL of ddH_2_O, 10 µL of SYBR® Premix Ex Taq II, 0.4 µL of ROX Reference Dye II, 0.4 µL of forward primer (10 µmol/L), 0.4 µL of reverse primer (10 µmol/L), and 2 µL of template cDNA. The PCR programs were run as follows: 5 min of pre-denaturation at 95°C, 40 cycles of 10 s at 95°C and 30 s at 60°C, followed by steps for dissociation curve generation (15 s at 95°C, 60 s at 60°C, and 15 s at 95°C). Dissociation curves for each amplicon were carefully examined to confirm the lack of multiple amplicons at different melting temperatures. Relative transcript levels for each sample were obtained using the comparative cycle threshold method using the cycle threshold value of Glyceraldehyde-3-phosphate dehydrogenase (GAPDH) gene as a control. The sequence of T7-2595, one of the unigenes annotated as GAPDH that exhibited no differences in expression among three tissues, was used for the primer design.

## Results

### RNA-seq and De novo Transcriptome Assembly

Illumina deep sequencing of the paper mulberry transcriptome yielded 54,638,676 clean reads, totaling 5.46 Gb. The sequencing data output and quality assessment are listed in Table S3. The average Q20 percentage (sequencing error rate, 1%), and GC percentage were 97.77% and 48.99%, respectively. Reads from three different tissues, namely root, stem and leaf, were individually assembled by Trinity. The resulting transcriptomes of the three tissues were 28.9, 25.0 and 19.8 Mb, respectively (Table S4). To acquire the comprehensive transcriptome of the paper mulberry, we complied 67,443 unigenes, of median length 723 bp, from the three individual tissue transcriptomes (Table S4). Prior to annotation and differential expression analysis, sequencing saturation was assessed to confirm that enough sequencing data had been obtained for further analysis. The results indicated that the sequencing data of three samples were sufficient for the follow-up expression analysis (Fig. S2). The assembled transcriptome data were deposited in NCBI’s Transcriptome Shotgun Assembly (TSA) database under the accession numbers from BP104589 to BP160056.

### Functional Annotation

The similarity search for all unigenes was performed against non-redundant protein sequences database (Nr) using BLASTX, and the annotation revealed that 37,725 unigenes encode products that have significant similarity to previously characterized proteins (E BL1e-5) (Table S5). Of the unigenes, 13,566 are longer than 1,000 bp. This sequence information can be used for direct amplification of the desired genes or cloning full-length genes by using methods like rapid amplification of cDNA ends (RACE) PCR.

**Figure 1:**
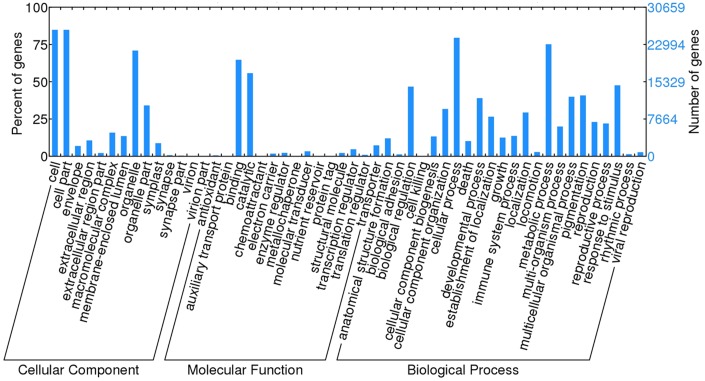
GO annotation of paper mulberry transcriptome. The results were summarized in three main categories: biological process, cellular component and molecular function. The right y-axis indicated the number of genes in a category. The left y-axis indicated the percentage of a specific category of genes in that main category. This was drawn using the online software and the URL was http://wego.genomics.org.cn/cgi-bin/wego/index.pl.

Gene ontology (GO) assignments were used to classify the functions of the predicted paper mulberry genes. Based on sequence homology, 30,659 sequences can be categorized into 63 functional groups (Fig. 1 and Table S5). In each of the three main categories (biological process, cellular component and molecular function) in the GO classification, “cellular process”, “cell part” and “binding” terms were dominantly marked, respectively. We also noticed a high percentage of genes from “metabolic process and response to stimulus”, “cell and organelle” and “catalytic” categories and only a few genes from the terms “protein tag”, “extracellular matrix part” and “cell killing”.

**Figure 2:**
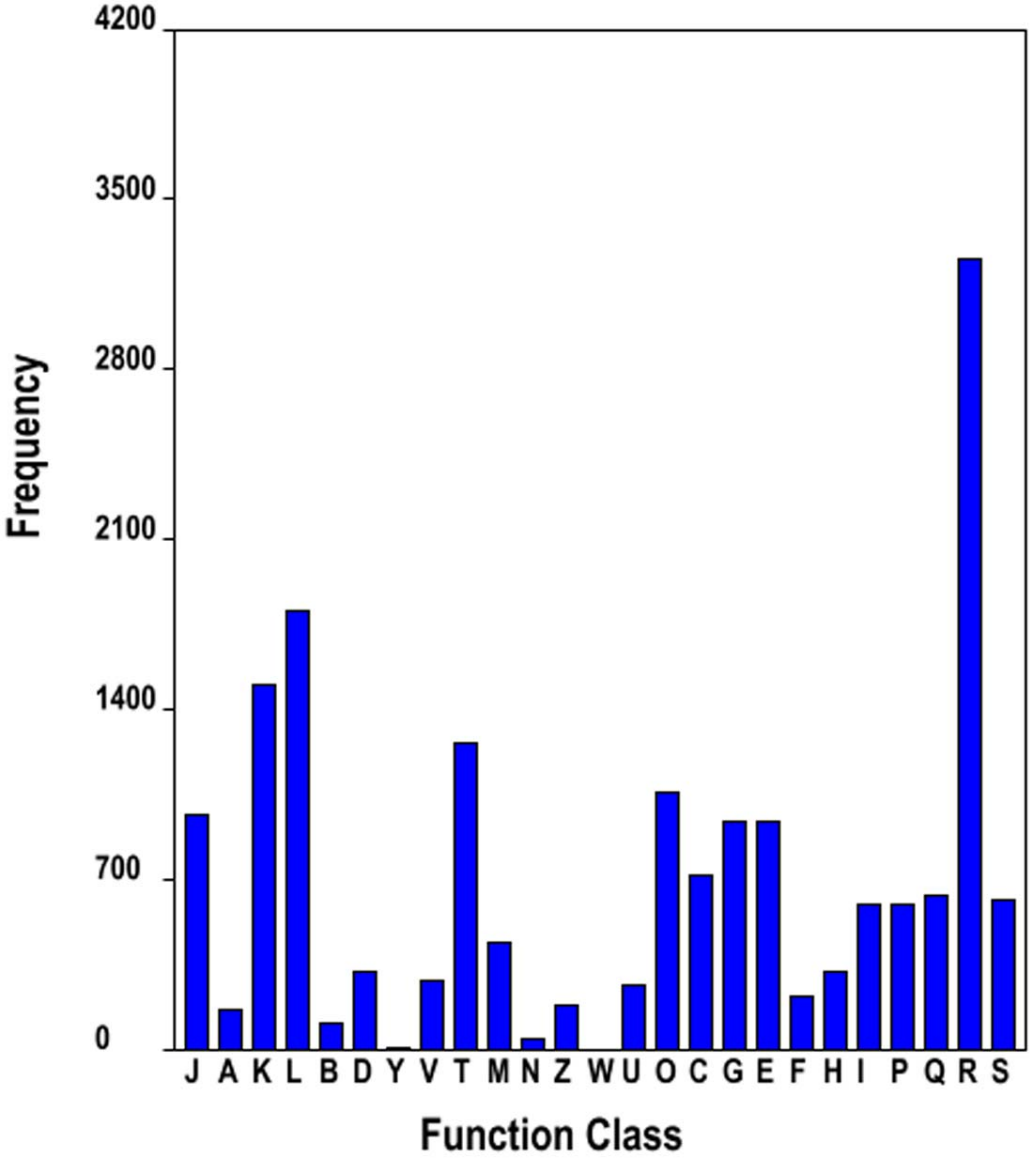
Clusters of orthologous groups (COG) of paper mulberry transcriptome. Out of 36,854 nr hits, 12,478 unigenes had a COG classification. J: Translation, ribosomal structure and biogenesis A: RNA processing and modification K: Transcription L: Replication, recombination and repair B: Chromatin structure and dynamics D: Cell cycle control, cell division, chromosome partitioning Y: Nuclear structure V: Defense mechanisms T: Signal transduction mechanism M: Cell wall/membrane/envelope biogenesis N: Cell mobility Z: Cytoskeleton W: Extracellular structures U: Intracellular trafficking, secretion, and vesicular transport O: Posttranslational modification, protein turnover, chaperones C: Energy production and conversion G: Carbohydrate transport and metabolism E: Amino acid transport and metabolism F: Nucleotide transport and metabolism H: Coenzyme transport and metabolism I: Lipid transport and metabolism P: Inorganic ion transport and metabolism Q: Secondary metabolites biosynthesis, transport and metabolism R: General function prediction only S: Function unknown.

To further characterize the comprehensive transcriptome of the paper mulberry, all of the unigenes were annotated by COG classifications. In total, out of 36,854 nr hits, 12,478 unigenes have a COG classification (Fig. 2 and Table S5). Among the 25 COG categories, the cluster for “general function prediction” represented the largest group (3,252, 18.92%) followed by “replication, recombination and repair” (1,803, 10.49%) and “transcription” (1,497, 8.71%). The categories of “nuclear structure” (5, 0.029%) and “cell motility” (78, 0.25%) represented the smallest groups.

**Figure 3:**
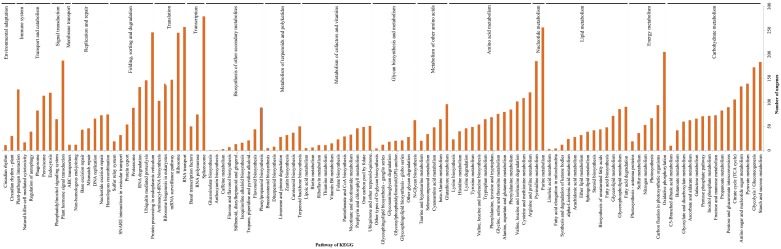
KEGG (Kyoto Encyclopedia of Genes and Genomes) annotation of paper mulberry transcriptome. The right y-axis indicated the number of unigenes in a category. The up x-axis showed the main cluster of KEGG pathway.

To identify the most enriched biological pathways in the three tissues of the paper mulberry assessed, we mapped the 30,659 annotated sequences to the referred canonical pathways in KEGG. We found that 7,199 sequences in total were directly associated to 119 KEGG pathways (Fig. 3 and Table S5). The annotated pathways included metabolism (4,788 unigenes), genetic information processing (2,328 unigenes), environmental information processing (264 unigenes), cellular processes (356 unigenes) and organismal systems (185 unigenes). In the primary metabolism, 1,182 unigenes mapped to amino acid metabolism. In the secondary metabolism subclass, 355 unigenes mapped to metabolism of terpenoids and polyketides, flavonoid biosynthesis and other secondary metabolites.

### Tissue-specific Transcriptome Analysis

**Figure 4:**
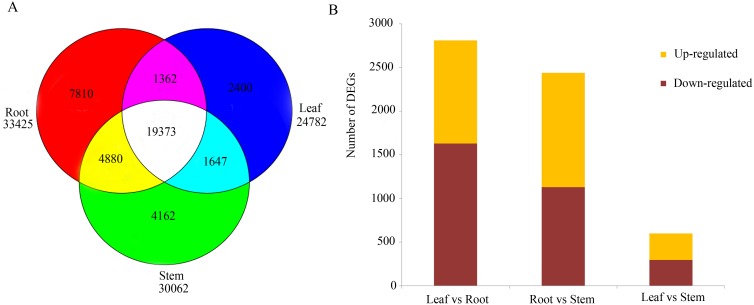
Unigenes expressed in different tissues of paper mulberry. A Venn diagram of unigenes expressed in different tissues of paper mulberry. B Differentially expressed unigene number among different tissues of paper mulberry. The number of up-regulated and down-regulated unigenes between root and leaf, root and stem, and stem and leaf were summarized. In this study, DEGs with higher expression levels in one tissue (such as root) when compared with another tissue (such as stem) were denoted as ‘up-regulated’, while those with lower expression levels were denoted as ‘down-regulated’. The unigene which Fold changee (such as root) when compared with another tissue (such as stem) were denoted.

Venn analysis of the unigenes expressed in three tissues of paper mulberry revealed that 19,373 unigenes are co-expressed and more unigenes were expressed specifically in the root than the other two tissues (Fig. 4A). To identify genes showing a significant expression difference among tissues, the RNA-seq data of three samples were processed by IDEG6 program. A total of 2,438 DEGs were detected between the root and the stem libraries. Among them 1,309 unigenes were up-regulated (meaning expressed higher in the root) and 1,129 unigenes were down-regulated (Fig. 4B, Table S6). Between the leaf and root, a total of 2,809 DEGs were detected with reduced up-regulated genes (1,181) and increased down-regulated genes (1,628) (Table S7). Between the stem and leaf, 600 DEGs were detected with 305 up-regulated genes and 295 down-regulated genes (Table S8). This suggested that, compared with the DEGs between stem and leaf, there were more DEGs between root and leaf. These RNA-seq results were verified by qPCR (Fig. S3).

### Identification of SSRs

A total of 11,801 SSRs were discerned from 7,029 unigenes of the paper mulberry (Table S9). There were 2,943 unigenes with more than 1 SSR and 1,276 SSRs presented in a compound formation manner (Table S9). Mononucleotide repeats made up the highest proportion of SSRs at a frequency of 54.27%, followed by di- (30.28%) and tetra- nucleotide (2.67%) repeats. The lowest frequency of SSRs was penta- and hexa-nucleotide repeats (Table S9). Motifs of AG/CT (2,422) were the predominant dinucleotide repeats. AAG/CTT (727) was the primary motif among the tri-nucleotide repeats.

## Discussion

### The First Accurately Assembled Transcriptome of the Paper Mulberry

Despite the important economic value of the paper mulberry, its transcriptomic and genomic data are not available in public databases. Illumina RNA-seq technology has been extensively used in the transcriptome sequencing for the model plants [33] with reference genome data or non-model plants [28] without reference genomic information. Here, we obtained for the first time the genome-wide expression profiling of the paper mulberry using the Illumina sequencing platform and the Trinity program. About 67,732 unigenes were accurately de novo assembled and 37,725 unigenes were successfully annotated via Blastx. Comparison of assembled unigenes to gene catalogs of other plant species by alignment and functional annotation indicated that the unigenes obtained in this study represent a large proportion of the genes expressed in the paper mulberry. The annotation against the NCBI Nr database showed that 10,026 unigenes were primarily matched with *Prunus perscia*, and followed by *Vitis vinifera* (5,925), *Fragaria vesca* (3,358), *Populus trichocarpa* (2,545), *Ricinus communis* (2,427), *Cucumis sativus* (1,498), *Glycine max* (1,214), *Medicago truncatula* (593), and so on (Fig. S4A). The paper mulberry belongs to the family Moraceae, *Prunus perscia* and *Fragaria vesc*a belong to Rosaceae, and all three belong to the order Rosales. Meanwhile, *Populus trichocarpa* belongs to the family Salicaceae and *Ricinus communis* belongs to Euphorbiaceae. The alignment data clearly correlate with the phylogenetic relationships among these species, with *Prunus perscia*, the closest relative of the paper mulberry, having the greatest number of first alignment hits. 22,692 paper mulberry unigenes aligned to sequences in the Nr data with more than 60% identity, which accounted for a large proportion of the total unigenes. The identity of 7,722 unigenes was more than 80% (Fig. S4B). Nearly half the unigenes (18,641) unigenes aligned with an E-value of less than 1e-50 (Fig. S4C). Those alignment results showed that the transcripts were correctly assembled and represented the actively expressed genes of the paper mulberry. Due to lack of genome and EST information in paper mulberry, 44.1% of unigenes could not be matched to known genes with a significant percentage of short sequences. Similarly, up to 38,617 transcripts (57.25% of total) had no Swissprot annotation. The statistical analysis of annotation was listed in Table 1.

**Table 1:** Statistic of functional annotation.

Annotation Database	Number	300< = length<1000	length> = 1000
COG	17184	4453	6010
GO	30659	12837	11977
KEGG	7199	2719	3326
Swissprot	29210	12184	11785
**N**r	36854	16382	13197
All	37725	16648	13202

### Cellulose and Lignin Biosynthesis of Paper Mulberry

The plant cell wall is a complex structure composed of cellulose, hemicellulose, lignin, pectin, and protein. The biosynthesis and modifications of lignin have a profound impact on plant growth and development and cell wall properties. The combined effects of COMT down-regulation and F5H over-expression have substantial effects on plant growth and architecture in Arabidopsis [34]. Cellulose is the key precursor for industrial applications, such as paper making [35]. That the abundant bast fiber of this paper mulberry is an outstanding raw material for pulping was recognized for papermaking since Cai Lun’s invention of paper centuries ago [4, 36 and 37]. Lignin and pectin are the main factors that determine the pulping quality of paper mulberry [38] as well as the digestion and nutrient absorption in livestock husbandry [39]. According to the function annotation, 2,029 unigenes were clustered into the cell wall term, with 1,243 unigenes assigned to the cell wall biosynthesis and 2,474 unigenes assigned to the biology process of cell wall organization (Fig. 6 and Table S10). The annotation results of KEGG demonstrated that 89 unigenes are involved in the phenylpropanoid biosynthesis pathway (Table S11) that is associated with the monolignol biosynthesis [40].

Of these genes, 12 (Table 2) map to PAL (Phenylalanine Ammonia-Lyase), the rate-limiting enzyme in the phenylpropanoid biosynthesis as well as the first crucial enzyme in monolignol biosynthetic pathway [41], [42]. In addition, F5H and CCoAOMT, formerly isolated by RACE-PCR and registered in Genbank, were also detected in this sequencing data. Caffeoyl shikimate esterase (CSE), an enzyme in the lignin biosynthetic pathway that, together with 4CL, bypasses the second HCT reaction can significantly affect the lignin content in plant [43]. Six unigenes were annotated as monoglyceride lipase, which is the ortholog of CSE in Arabidopsis (Table S5).

**Table 2:** Identification of lignin biosynthesis genes.

Enzymes	Numbers of genes				
	Paper mulberry	Poplar*	Arabidopsis*	Medicago*	Rice*
C3H	1	4	3	1	1
F5H	1	4	2	3	3
C4H	4	3	1	1	4
4CL	26	22	13	10	16
CAD	6	21	9	21	5
HCT	12	7	1	6	9
CCOAMT	3	7	7	4	11
CCR	14	40	7	18	55
PAL	12	6	4	4	14
COMT	24	35	16	26	38

Abbriviations: CAD: Cinnamyl alcohol dehydrogenase, PAL: L-Phenylalanine ammonia-lyase, CCOAMT: Cinnamoyl CoA O-methyltransferase, COMT: Caffeic acid O-methyltransferase, 4CL: 4-Coumarate coenzyme A (CoA) ligase, CCR: Cinnamoyl-CoA reductases, C4H: Cinnamate-4-hydroxylase, HCT: Hydroxycinnamoyl transferase, C3H: 4-Coumarate 3-hydroxylase, F5H: Ferulate 5-hydroxylase.

*Those data were referenced from Xu et al., 2009.

**Figure 5:**
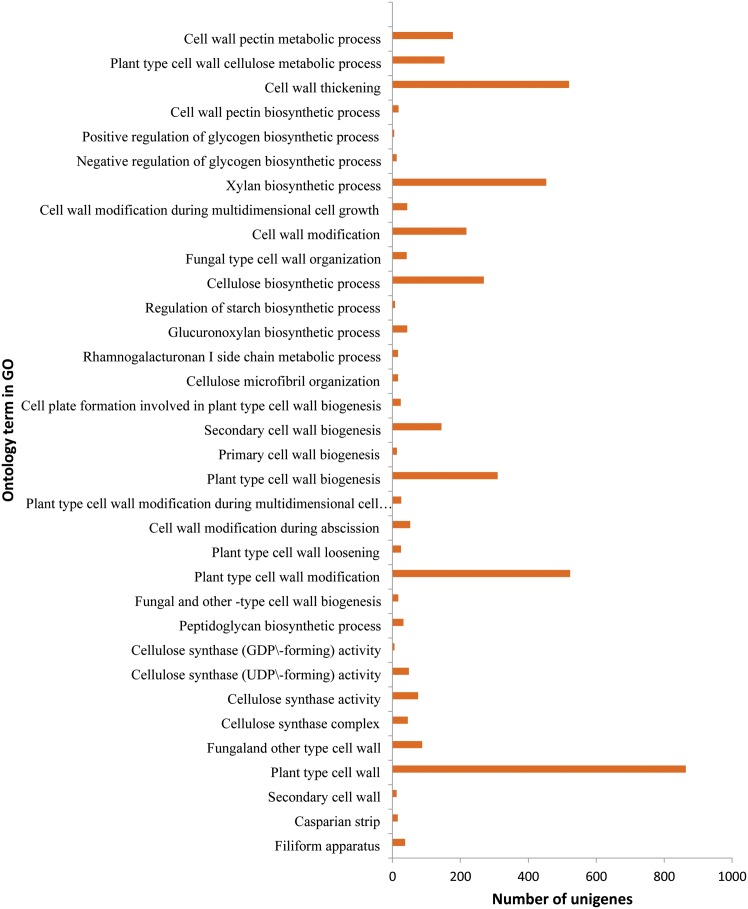
Cell wall related ontology in GO annotation. The unigenes related to cell wall were listed separately according to the GO term. This was drawn using the online software and the URL was http://wego.genomics.org.cn/cgi-bin/wego/index.pl.

Cellulose is synthesized by a large multimeric cellulose synthase (CesA) [35]. In the paper mulberry transcriptome, 45 unigenes mapp to cellulose synthase A (Table S5 and S10). There are 129 and 293 unigenes mapped to the cellulose synthase activity of molecular function and cellulose biosynthetic process of biological process in GO, respectively (Fig. 5 and Table S10). Xyloglucan is the most abundant hemicellulose in the cell wall of the dicotyledonous [44] and our study identified 43 unigenes as candidates in the xyloglucan biosynthesis pathway of the paper mulberry. They include 24 xyloglucan endotransglycosylase (XET or XTH) genes, 11 xyloglucan galactosyl transferase genes, xylose isomerase and so on (Table S5). All of these are certainly important targets for genetic manipulation of the lignin biosynthetic pathway.

**Figure 6:**
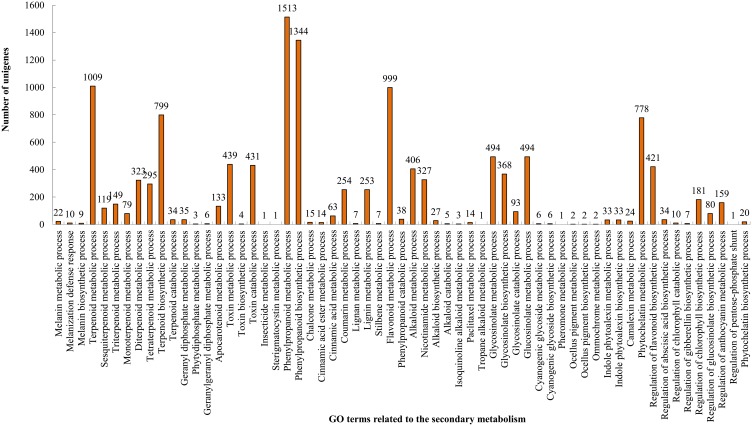
GO terms related to the secondary metabolism.

### Genes Related to Flavonoid and Other Secondary Metabolism

Another striking feature of the enriched GO and KEGG categories is that these genes are preferentially clustered in pathways relating to the biosynthesis of flavonoids and other secondary metabolites. Flavonoids are a major class of plant secondary metabolite to serve a multitude of functions, including pigmentation and antioxidant activity. Recent studies indicate that prenylated flavonols isolated from paper mulberry also have highly effective inhibition of alpha-glucosidase activity [45]. The flavenoid broussochalcone A is the principal inhibitor of cholinesterase [46], tyrosinase and xanthine oxidase [47] and also exhibits potent anti-tumor activity [48]. Therefore, identification of these genes has important implications in the eventual better understanding of the biosynthesis of flavonoids, which may be potent components of drug treatments of human conditions.

A total of 3,347 unigenes falling under 61 GO terms included in the secondary metabolic process could be identified in the GO annotation results (Fig. 6 and Table S12). Phenylpropanoid metabolic and biosynthesis process, the upstream pathway of flavonoids biosynthesis, contained a large number of unigenes (1,513) and 1,344 additional unigenes mapped to secondary metabolic processes. 1,009 and 406 unigenes were annotated as terpenoid metabolic process and alkaloid metabolic process, respectively. In addition, 999 unigenes were mapped to the flavonoids synthesis pathway (Fig. 6). Flavonoids are synthesized from phenylpropanoid derivatives by condensation with malonyl-CoA. Unigenes for CHS (Chalcone synthase), CHI (chalcone isomerase), F3H (flavanone 3-hydroxylase) and other enzymes involved in flavonol biosynthesis were mapped in the KEGG pathway (Fig. S5, S6 and S7; Table S5). Dihydroquercetin or dihydrokaempferol is the common intermediate for anthocyanidins and flavonols. DFR (dihydroflavonol 4-reductase) and flavonol synthase compete for the same substrate to drive the metabolism either to anthocyanidin biosynthesis or to flavonol biosynthesis [44]. One *DFR* gene was also identified from the sequencing data (Fig. S5). These sequences provide useful information for elucidation in the remaining steps and exploring the flavonoid biosynthetic pathway of the paper mulberry. In addition to flavonoid biosynthesis, 62 unigenes mapped to the alkaloid, stilbenoid and anthocyanin biosynthesis pathways (Fig. S8, S9, S10 and S11; Table S11).

### Biotic and Abiotic Stress Defensive Process in Paper Mulberry

**Figure 7:**
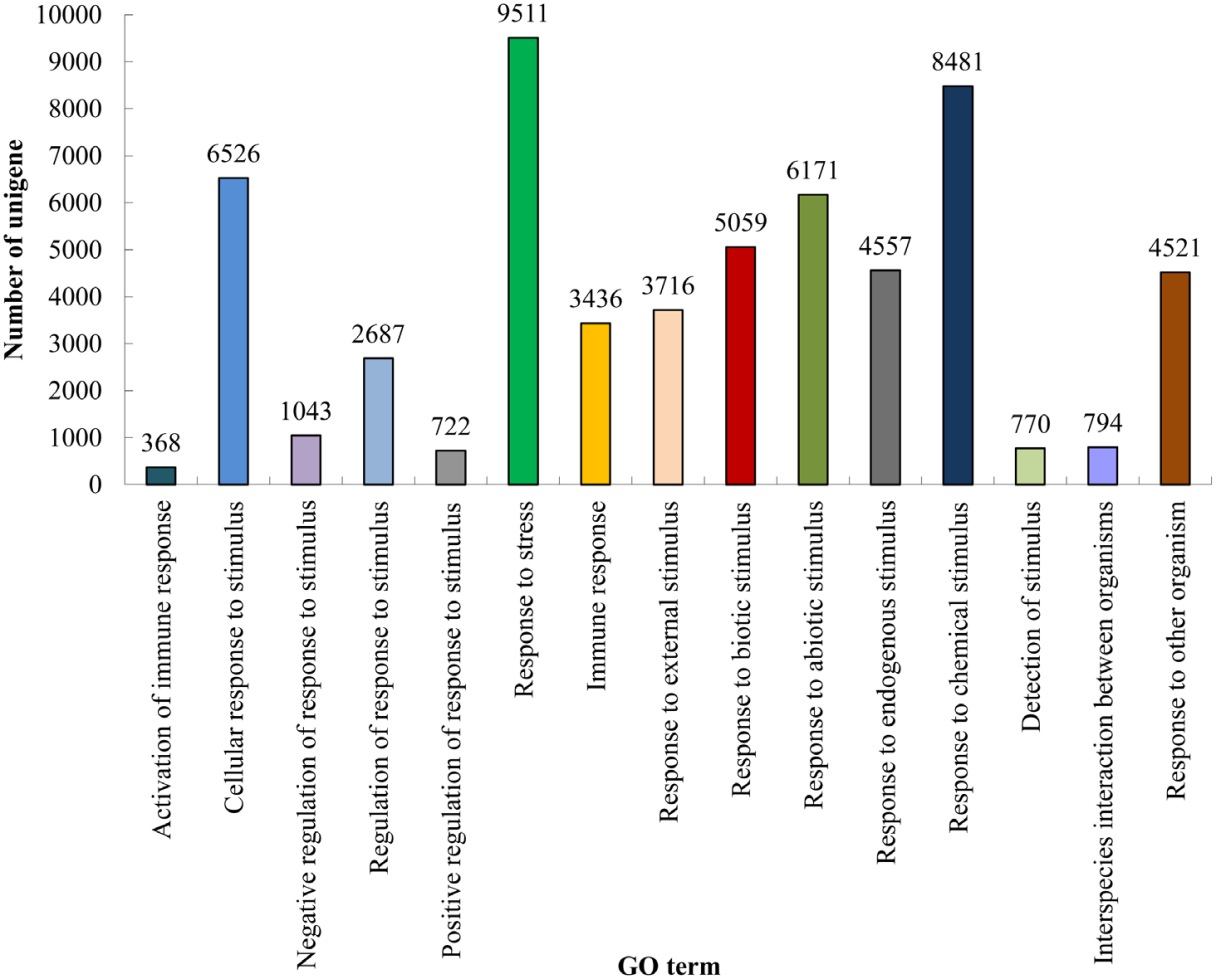
GO terms related to the stress response.

Understanding biotic and abiotic stress responses in plants is an important and challenging topic in plant research. Many biotic and abiotic stress-inducible genes were isolated and their functions were precisely characterized in transgenic plants in the past decade [49]. Integrated data obtained with various ‘omics’ approaches have generated a more comprehensive picture of abiotic stress responses. Therefore, additional transcriptomics research on stress responses will increase our knowledge regarding the adaptive mechanisms of paper mulberry in nature. Our results from the GO annotation showed that 6,171 and 5,059 unigenes were clustered in responsive to abiotic and biotic stimuli, respectively (Fig. 7, Table S13). These results provide reasonable explanations for the strong adaptability and tolerance of paper mulberry to unfavorable environmental conditions [1], [2] and the interfered development of *P. xylostella* population and insect pest control by ethanol extracts of paper mulberry [50]. Among these unigenes, 47 were the glucan endo-1, 3-beta-glucosidase that functions in the lysis of cell walls containing beta-1, 3-glucans, implicating their roles in the defense against fungal pathogens. Protein kinases also play important roles in plant development and response to various unfavorable environmental conditions. GsSRK (G-type lectin S-receptor-like serine/threonine protein kinase), a protein kinase, is required for plants to respond to abiotic and biotic stresses [51]. There were 243 unigenes annotated as GsSRK in the paper mulberry transcriptome, which might confer faster and stronger adaptation to external stimuli upon the paper mulberry. In addition, 45 glutathione S-transferase, 4 glutathione peroxidase, 3 glutathione reductases and 5 glutathione gamma-glutamylcysteinyl transferases, as well as many other detoxification-related genes were found in the paper mulberry transcriptome.

Importantly, 22 unigenes were heavy metal-associated and 778 unigenes (Fig. 6 and Table S12) were mapped to the phytochelatin metabolic process. Phytochelatins play an important role in the detoxification of heavy metals in plants [52]. This was consistent with previous reports that the leaf powder of paper mulberry was a great choice as a metal adsorbent [17]. Our findings might explain why the paper mulberry is used as the pioneer species for ecological afforestation and greenification of both sides of highways and mined land areas [1].

### Differentially Expressed Genes in Root, Stem and Leaf

According to the results of the DEG analysis (Fig. 4B), most DEGs were expressed in both roots and leaves. Leaves are mature organs, while roots and stems have more physical and molecular process for the elongation, differentiation and other functions. This dichotomy may explain why relatively fewer genes (24,782) are expressed in the leaf vs. the other tissues analyzed (Fig. 4A).

**Figure 8:**
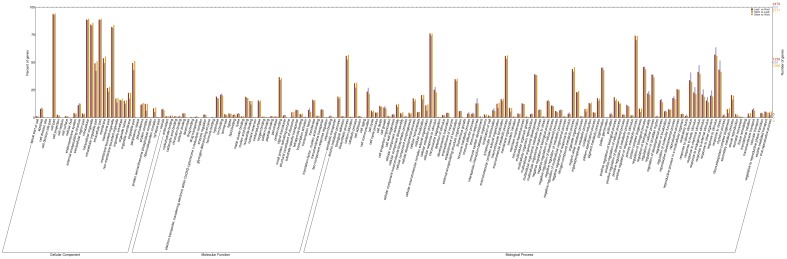
GO annotation of DEGs among different tissues of paper mulberry.

**Figure 9:**
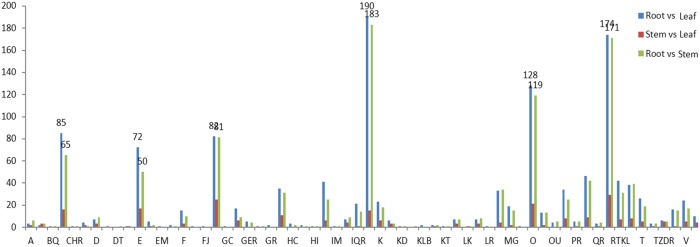
COG annotation of DEGs among different tissues *of* paper mulberry. The enriched orthologous were as follows: J: Translation, ribosomal structure and biogenesis O: Posttranslational modification, protein turnover, chaperones C: Energy production and conversion G: Carbohydrate transport and metabolism E: Amino acid transport and metabolism R: General function prediction only.

Genes with similar expression patterns may function in the same process. The association between GO terms and the lists of DEGs was tested using the GO functional enrichment analysis. In GO terms, cell component, cell part, membrane and membrane-bounded organelle were significantly enriched in DEGs between the root vs. leaf and root vs. stem (Fig. 8). Interestingly, no significantly enriched GO terms were found in genes with differential expression between leaves and stems. Among biological processes, cellular metabolic process contained the most unigenes, and the top three classes of genes identified were primary metabolic process, macromolecular metabolic process and response to stimulus. As for COG analysis, most DEGs were clustered in translation and ribosomal structure and biogenesis, including posttranslational modification; protein turnover; chaperones; carbohydrate transport and metabolism; energy production; and conversion; and amino acid transport and metabolism (Fig. 9).

**Figure 10:**
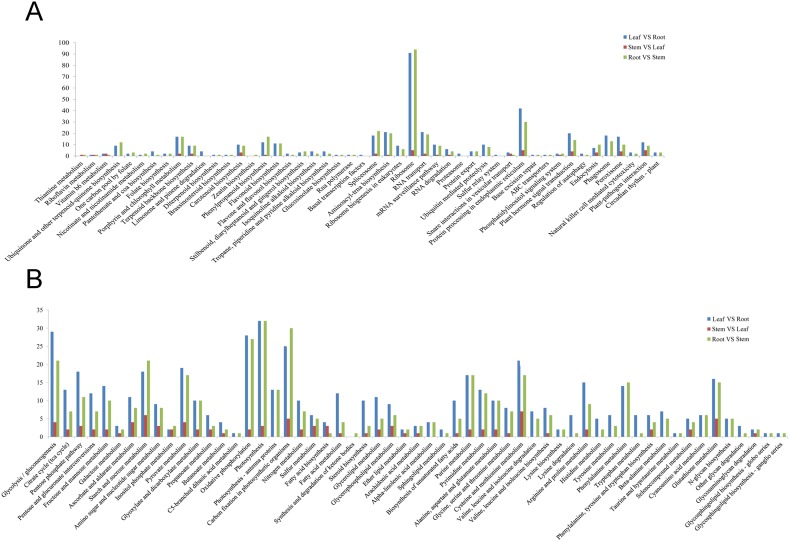
KEGG annotation of DEGs among different tissues of paper mulberry.

Pathway enrichment analysis was performed to further connect the biological functions of DEGs. We mapped all DEGs to terms in the KEGG database as compared to the whole transcriptome background, expecting significant enrichment of genes involved in metabolic or signal transduction pathways in the DEGs. Indeed, 101 pathways were specifically enriched in leaves vs. roots (Fig. 10 A and B; Table S11); these are primarily involved in three pathways: ribosome, biosynthesis of secondary metabolites, and photosynthesis. Other enriched pathways include phenylpropanoid biosynthesis, phenylalanine metabolism, and flavonoid biosynthesis. Interestingly, genes belonging to 102 pathways were found to be enriched in the root vs. the stem. These genes are primarily involved in protein processing in three pathways of endoplasmic reticulum, ribosome and photosynthesis. Base on the percentage of all unigenes, DEGs are mainly found in the translation and photosynthesis. By contrast, few DEGs are related to post transcription modifications.

According to the DEGs annotation results (Fig. 8 and table S6, S7 and S8), the expression of unigenes for photosynthesis were significantly more highly expressed in leaf and stem than in root. Some unigenes encoding the 30S and 50S ribosomal protein subunits are markedly increased in the leaves and stems vs. the roots, whereas those unigenes encoding the 40S and 60S ribosomal protein subunits are more highly expressed in the roots than that in leaves. Our results are consistent with the fact that the leaf is the main photosynthetic organ, though, interestingly the expression profile of some genes involved in photosynthesis is comparable in stems and roots, as exemplified by the expression of thylakoid luminal protein, pentatricopeptide repeat-containing protein and ribulose bisphosphate carboxylase/oxygenase activase. Meanwhile, many cell wall syntheses related genes including caffeic acid 3-O-methyltransferase, vegetative cell wall protein and cinnamyl alcohol dehydrogenase are highly expressed in the root (Fig. S12). Three cellulose synthase unigenes have increased expression in the leaf than that in the root, suggesting that these genes may contribute to mediation of the cellulose content of the paper mulberry leaf, which has implications for the use of this plant as a source of livestock and poultry feed. Regarding secondary metabolism, the expression level of two theobromine synthase, 1 tricyclene synthase, 4 chalcone synthase and 3 myrcene synthase unigenes is much higher in leaf than in root (Fig. S13 and Table S14), which highlights that some secondary metabolites are primarily synthesized in the leaf.

The majority of the DEGs are significantly up-regulated in the root vs. the leaf and stem. In contrast, no specific enrichment of genes was observed for any pathway in the comparison of the leaf and the stem. These results suggest that the secondary metabolism pathway might be more active in the root to provide a larger repertoire of secondary metabolites involved in physiological defense mechanisms. It also indicates that at the preliminary stage of organ differentiation, the genes expressed in the plantlet mainly functioned for morphogenesis.

## Conclusion

In this study, we have identified a large number of candidate genes that may be associated with the economically beneficial characteristics of the paper mulberry. Among of those annotated genes, 2,474 unigenes are involved in the biosynthesis of cellulose, pectin and lignin, and 800 unigenes are related to phytochelation metabolisms, are thus may make important contributions to our understanding of the growth of paper mulberry in heavy metal-contaminated soil. Our findings identified 5,059 genes for biotic stress response, 6,171 genes for abiotic stress response and 3,347 genes for flavonoids biosynthesis, which might contribute to the strong adaptability of the paper mulberry. The information about these genes provides us potent and valuable guidance in the development of new cultivars for the exploitation of the paper mulberry.

## Supporting Information

Figure S1 **The pipeline of the transcriptome experimental and bioinformatics analysis.**
(TIF)

Figure S2 **Sequencing saturation analysis of expression profile in three different tissues of paper mulberry.**
(TIF)

Figure S3 **Results of RNA-seq and Q-PCR validation.** The red and blue columns represented the results of RNA-seq and Q-PCR, respectively.
(TIF)

Figure S4 **The statistics of blast and alignment.** A Species statistic of unigene matched with in Nr database. B Identity analysis of unigene alignment. C The unigene E-value of alignment.
(TIF)

Figure S5 **KEGG pathway of flavonoid biosynthesis.**
(TIF)

Figure S6 **KEGG pathway of isoflavonoid biosynthesis.**
(TIF)

Figure S7 **KEGG pathway of flavone and flavonol biosynthesis.**
(TIF)

Figure S8 **KEGG pathway of anthcoyanin biosynthesis.**
(TIF)

Figure S9 **KEGG pathway of bstilbenoid, diarylheptanoid and gingerol biosynthesis.**
(TIF)

Figure S10 **KEGG pathway of isoquinoline alkaloid biosynthesis.**
(TIF)

Figure S11 **KEGG pathway of tropane, piperidine and pyridine alkaloid biosynthesis.**
(TIF)

Figure S12 **Lignin biosynthesis pathway of DEGs between leaf and root.** The gray background was the unigene that was detected in this study. The green background was the unigene that was higher expressed in root than that in leaf. They were listed as follows: [EC:1.14.13.11] trans-cinnamate 4-monooxygenase [EC:6.2.1.12] 4-coumarate–CoA ligase [EC:1.1.1.195] cinnamyl-alcohol dehydrogenase [EC:1.11.1.7] peroxidase.
(TIF)

Figure S13 **Flavonoid biosynthesis pathway of DEGs between leaf and root.** [EC:2.3.1.74] chalcone synthase [EC:1.14.11.9] naringenin 3-dioxygenase [EC:1.14.13.21]flavonoid 3′-monooxygenase [EC:1.14.13.11]trans-cinnamate 4-monooxygenase [EC:1.17.1.3] leucoanthocyanidin reductase [EC:1.14.11.19] leucoanthocyanidin dioxygenase [EC:1.3.1.77] anthocyanidin reductase.
(TIF)

Table S1 **Genes chosen for validation.**
(DOCX)

Table S2 **Primers for PCR.**
(DOCX)

Table S3 **The sequencing data output and quality assessment on the RNA-seq results of paper mulberry.**
(DOCX)

Table S4 **The assembled unigenes results of the RNA-seq results of paper mulberry.**
(DOCX)

Table S5 **Integrated function annotation of paper mulberry transcriptome.**
(XLSX)

Table S6 **DEGs of Root vs leaf.**
(XLSX)

Table S7 **DEGs of Stem vs root.**
(XLSX)

Table S8 **DEGs of Stem vs leaf.**
(XLSX)

Table S9 **SSR Statistic of paper mulberry transcriptome.**
(XLSX)

Table S10 **Cell wall related ontology in GO annotation.**
(XLSX)

Table S11 **KEGG annotation of paper mulberry transcriptome.**
(XLSX)

Table S12 **GO terms related to the secondary metabolism.**
(XLSX)

Table S13 **GO terms related to the stress response.**
(DOCX)

Table S14 **All of the DEGs and their annotation results of paper mulberry.**
(XLSX)
