# Correction: *De Novo* Assembly of Expressed Transcripts and Global Transcriptomic Analysis from Seedlings of the Paper Mulberry (*Broussonetia kazinoki* x *Broussonetia papyifera*)

**DOI:** 10.1371/journal.pone.0108613

**Published:** 2014-09-12

**Authors:** The PLOS ONE Staff

There are errors in the accession numbers in the Materials and Methods and Results.

The last sentence of the cDNA Library Preparation and Sequencing (RNA-seq) section of the Materials and Methods should read: Raw sequence data were generated by the Illumina pipeline and are available in NCBI’s Short Read Archive (SRA) database (http://www.ncbi.nlm.nih.gov/Traces/sra/sra.cgi) under accession number SRP029966.

The last sentence of the RNA-seq and De novo Transcriptome Assembly section of the Results should read: The transcriptome data were deposited in NCBI's Short Read Archive (SRA) database under accession number of SRP029966.
