# Supplementary figures and images for: De Novo Assembly of Expressed Transcripts and Global Transcriptomic Analysis from Seedlings of the Paper Mulberry (Broussonetia kazinoki x Broussonetia papyifera)
Source: PLoS One. 2014 May 21;9(5):e97487. doi: 10.1371/journal.pone.0097487 (PMC4029624; doi:10.1371/journal.pone.0097487)

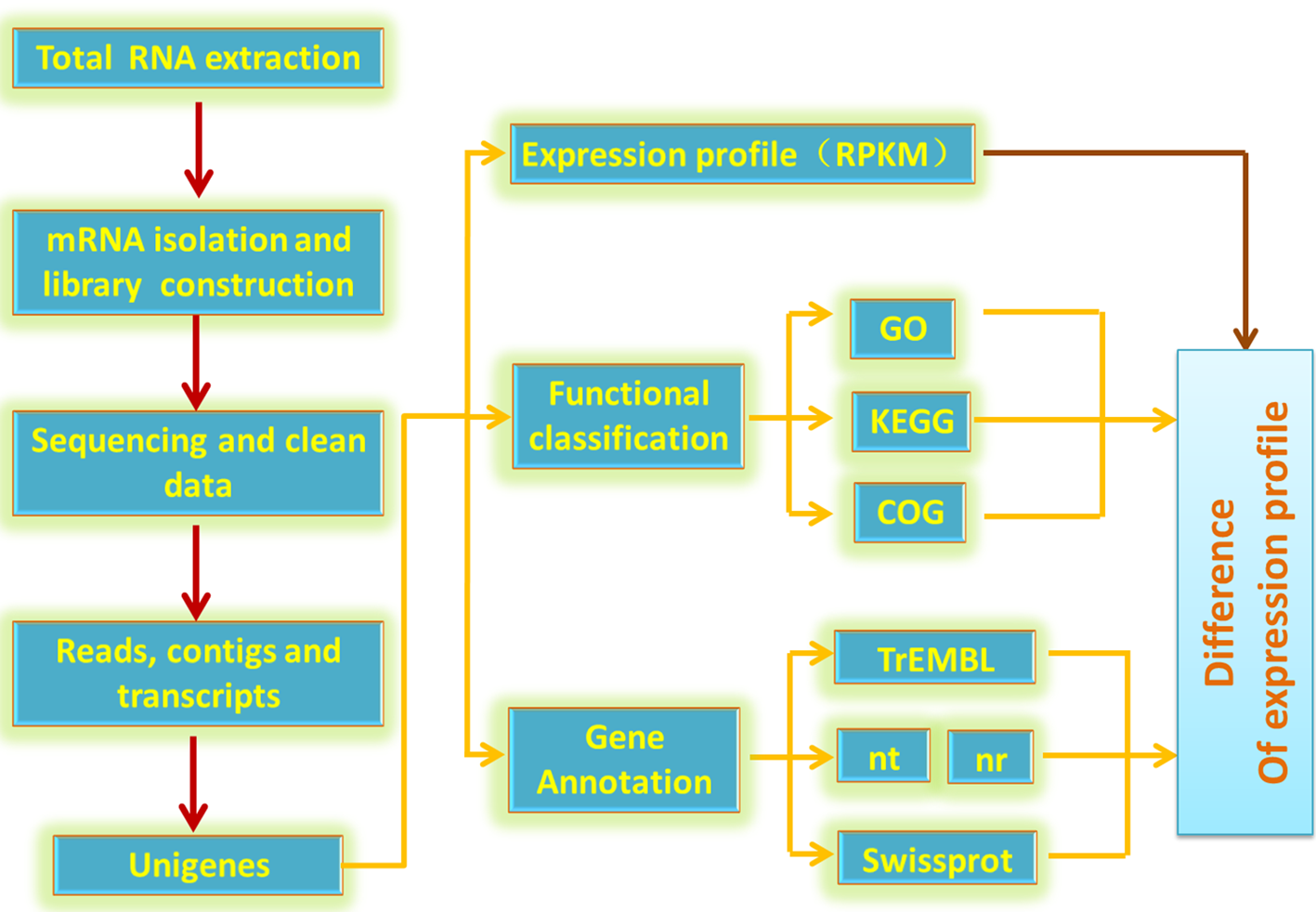

Supplement: Figure S1 — The pipeline of the transcriptome experimental and bioinformatics analysis. (TIF) [file pone.0097487.s001.tif]

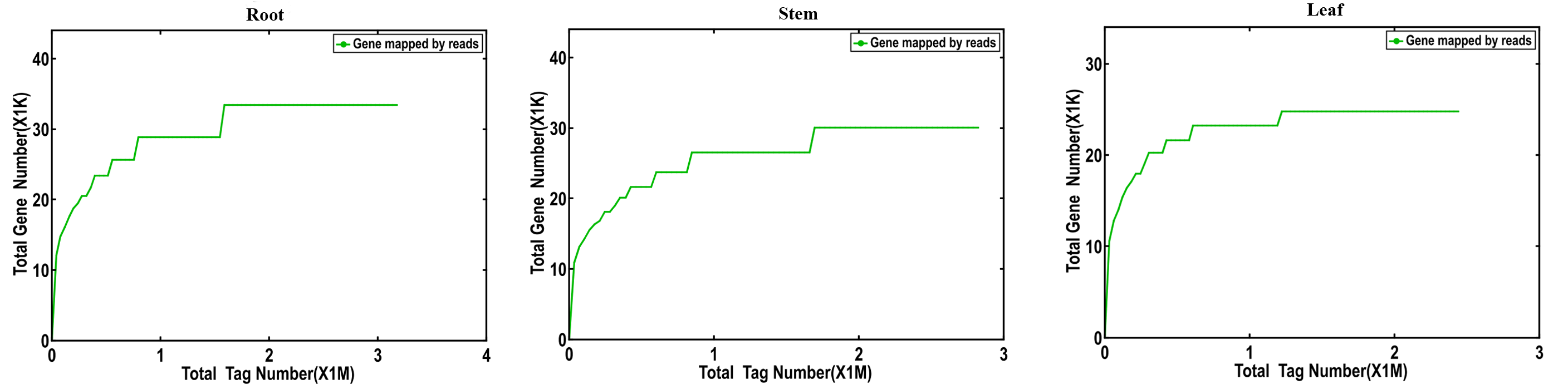

Supplement: Figure S2 — Sequencing saturation analysis of expression profile in three different tissues of paper mulberry. (TIF) [file pone.0097487.s002.tif]

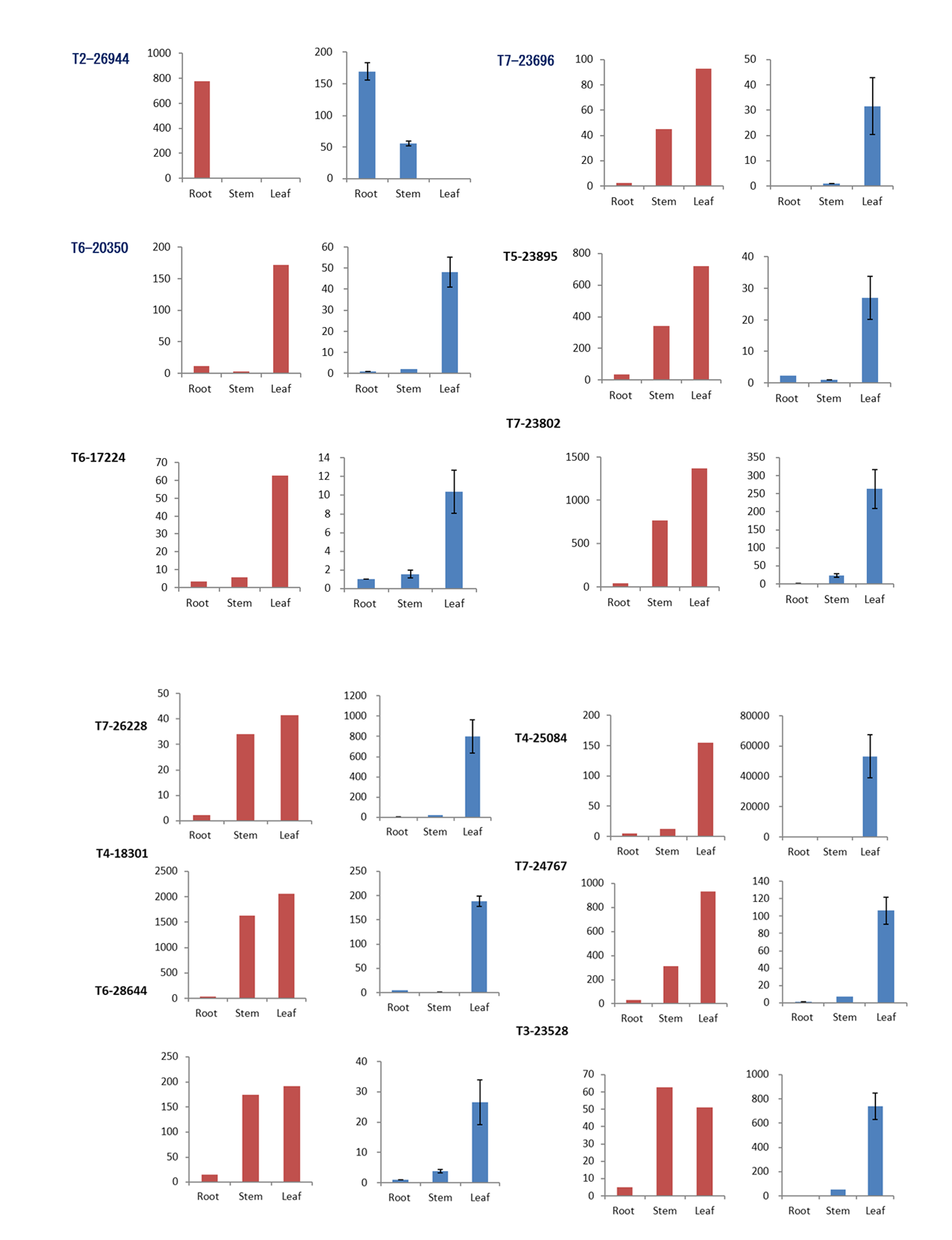

Supplement: Figure S3 — Results of RNA-seq and Q-PCR validation. The red and blue columns represented the results of RNA-seq and Q-PCR, respectively. (TIF) [file pone.0097487.s003.tif]

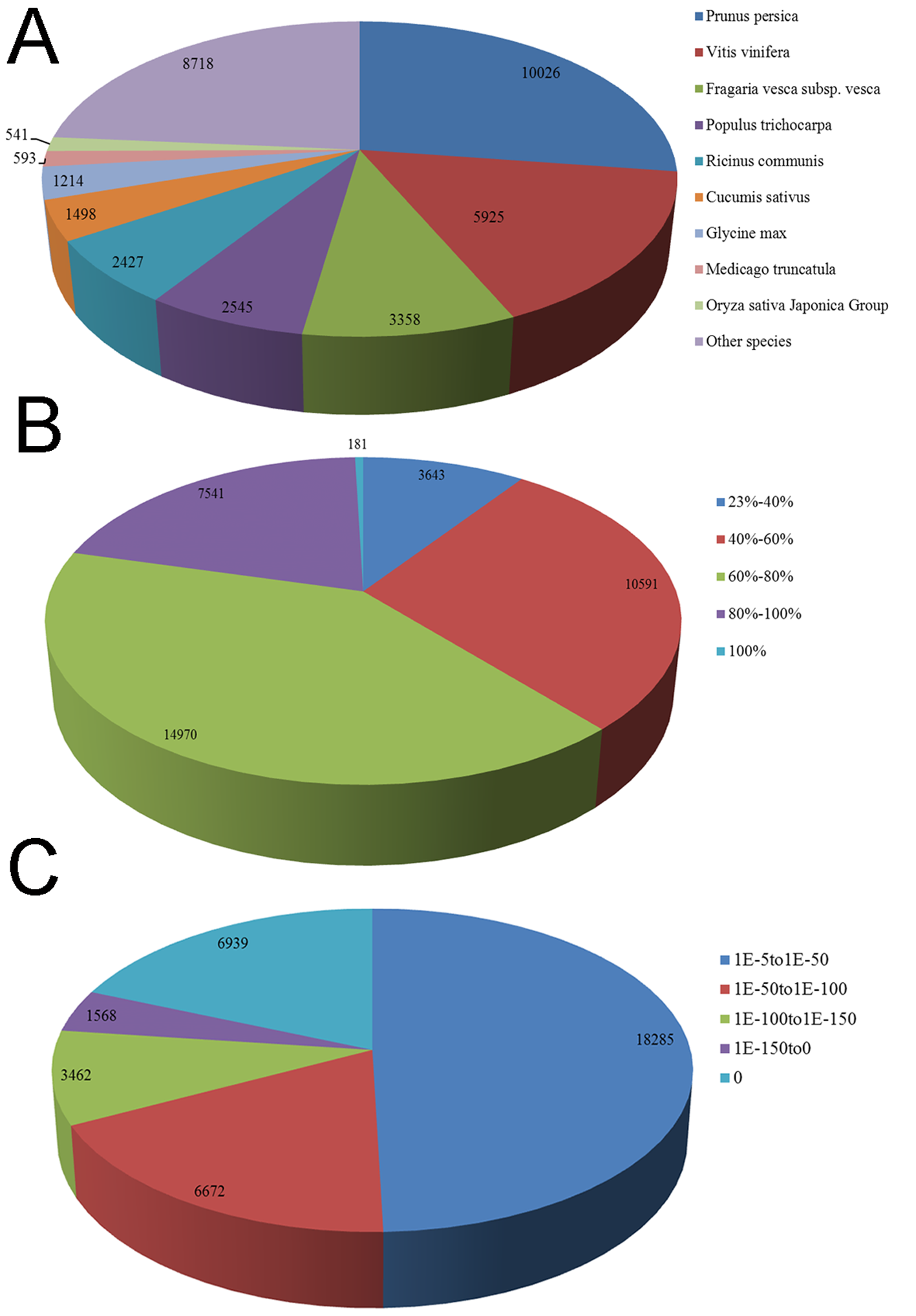

Supplement: Figure S4 — The statistics of blast and alignment. A Species statistic of unigene matched with in Nr database. B Identity analysis of unigene alignment. C The unigene E-value of alignment. (TIF) [file pone.0097487.s004.tif]

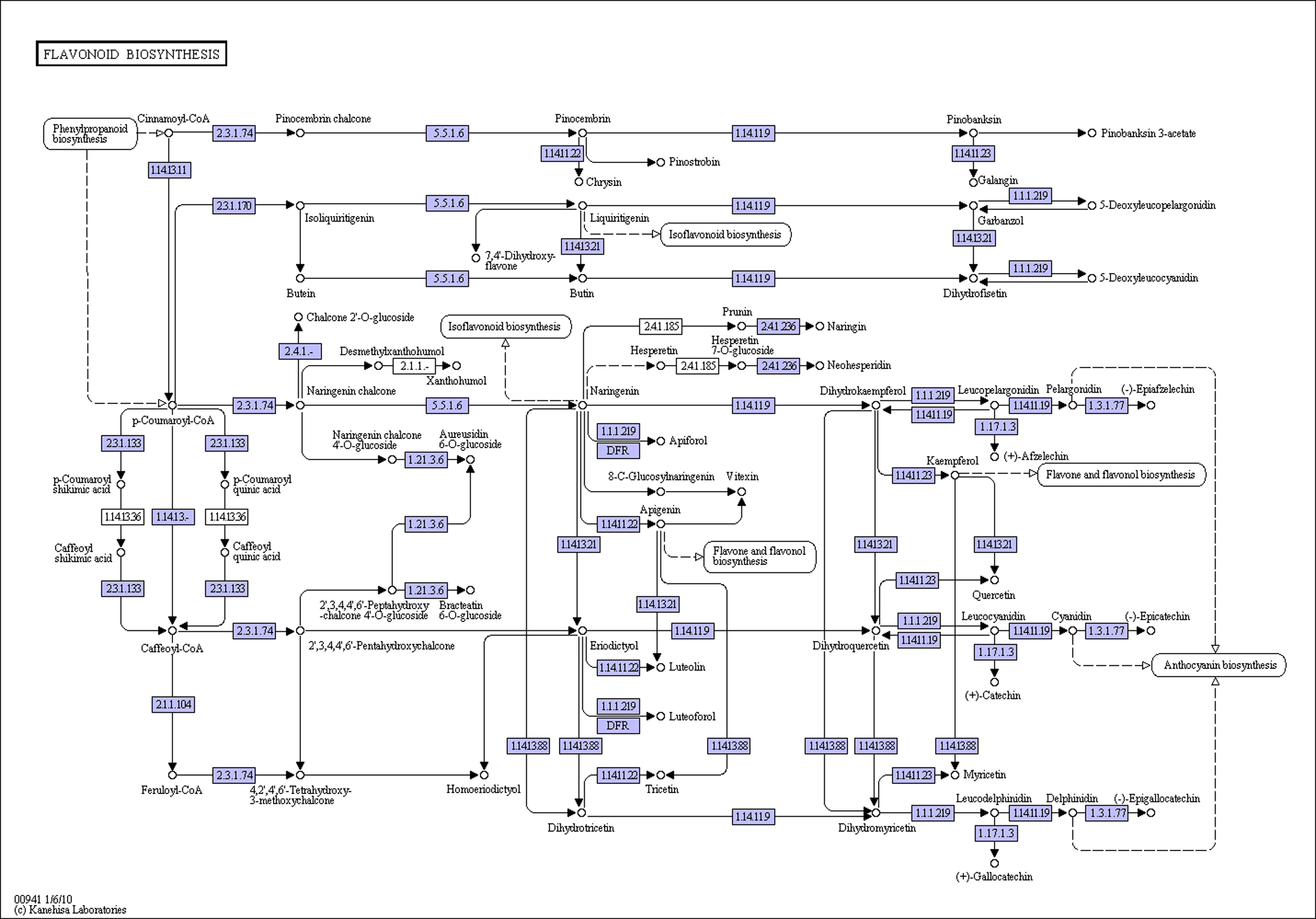

Supplement: Figure S5 — KEGG pathway of flavonoid biosynthesis. (TIF) [file pone.0097487.s005.tif]

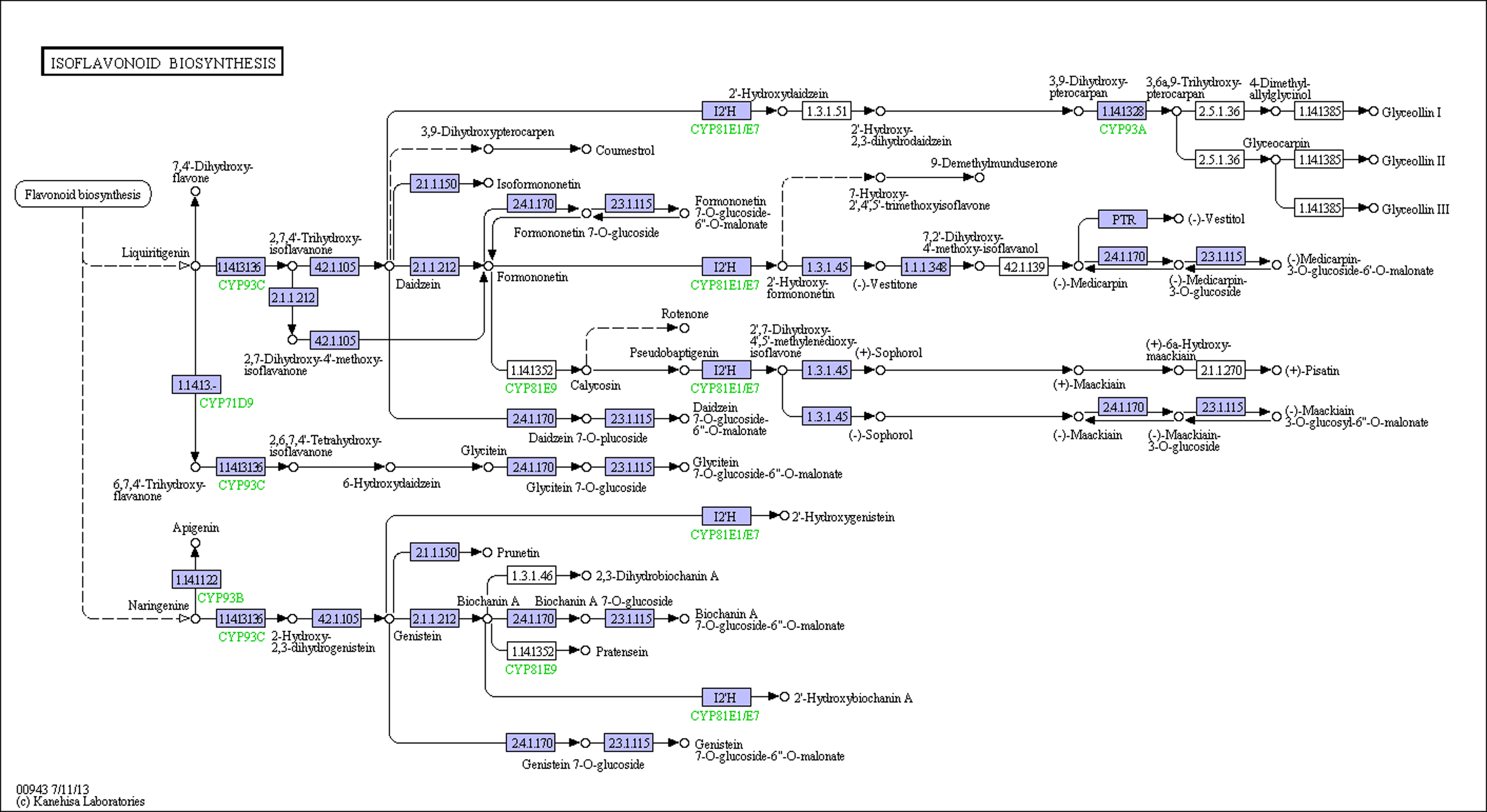

Supplement: Figure S6 — KEGG pathway of isoflavonoid biosynthesis. (TIF) [file pone.0097487.s006.tif]

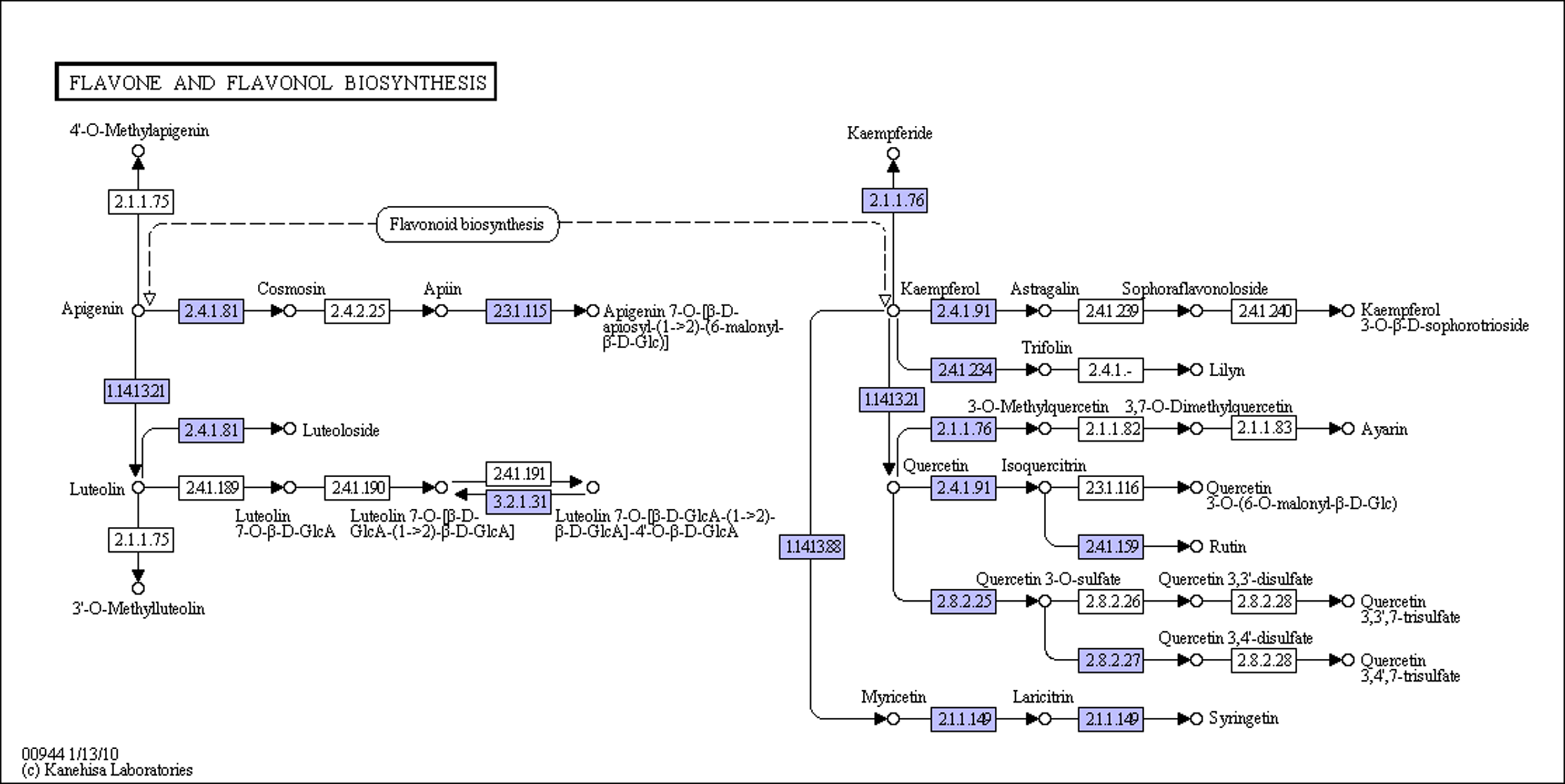

Supplement: Figure S7 — KEGG pathway of flavone and flavonol biosynthesis. (TIF) [file pone.0097487.s007.tif]

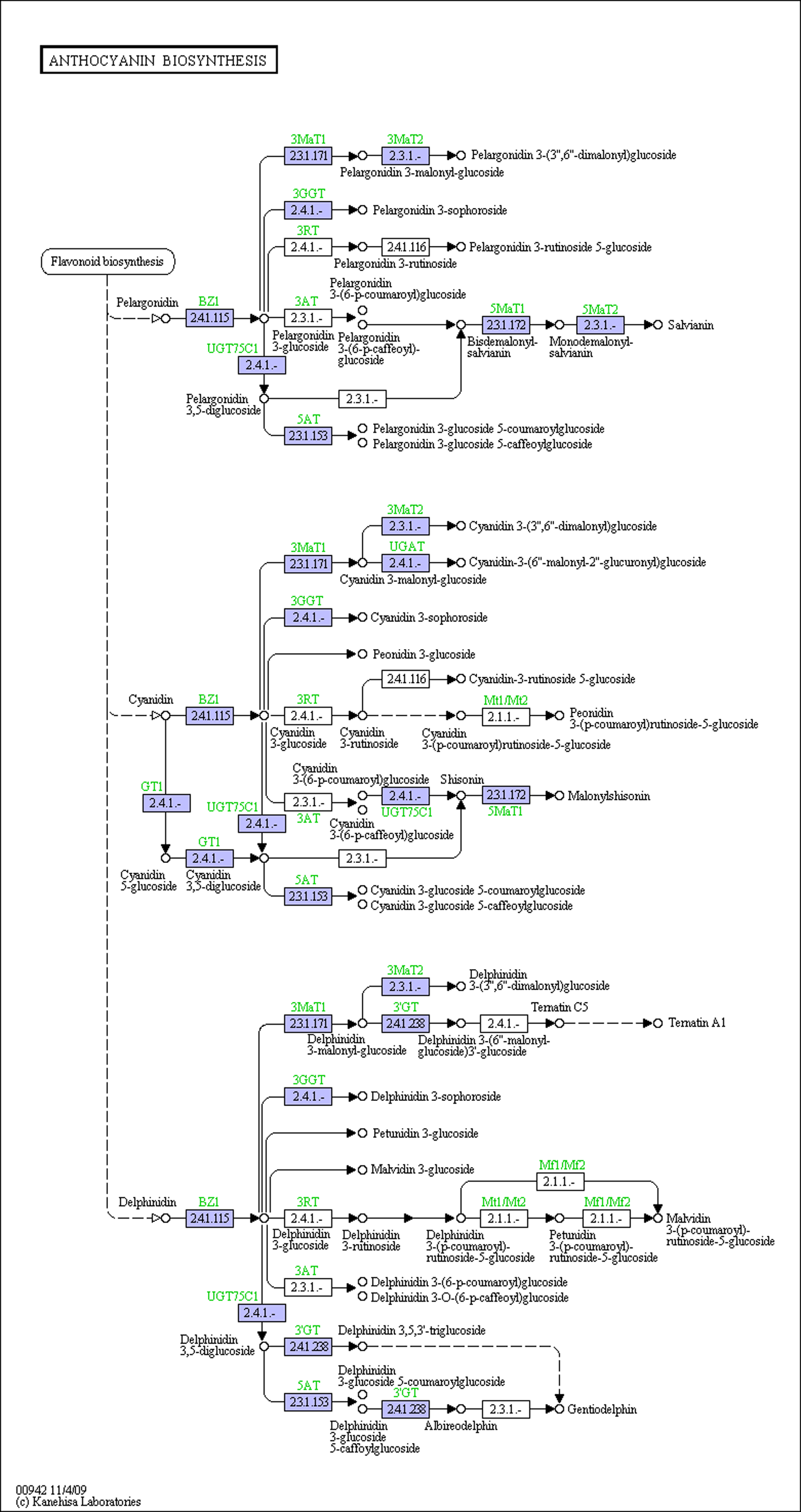

Supplement: Figure S8 — KEGG pathway of anthcoyanin biosynthesis. (TIF) [file pone.0097487.s008.tif]

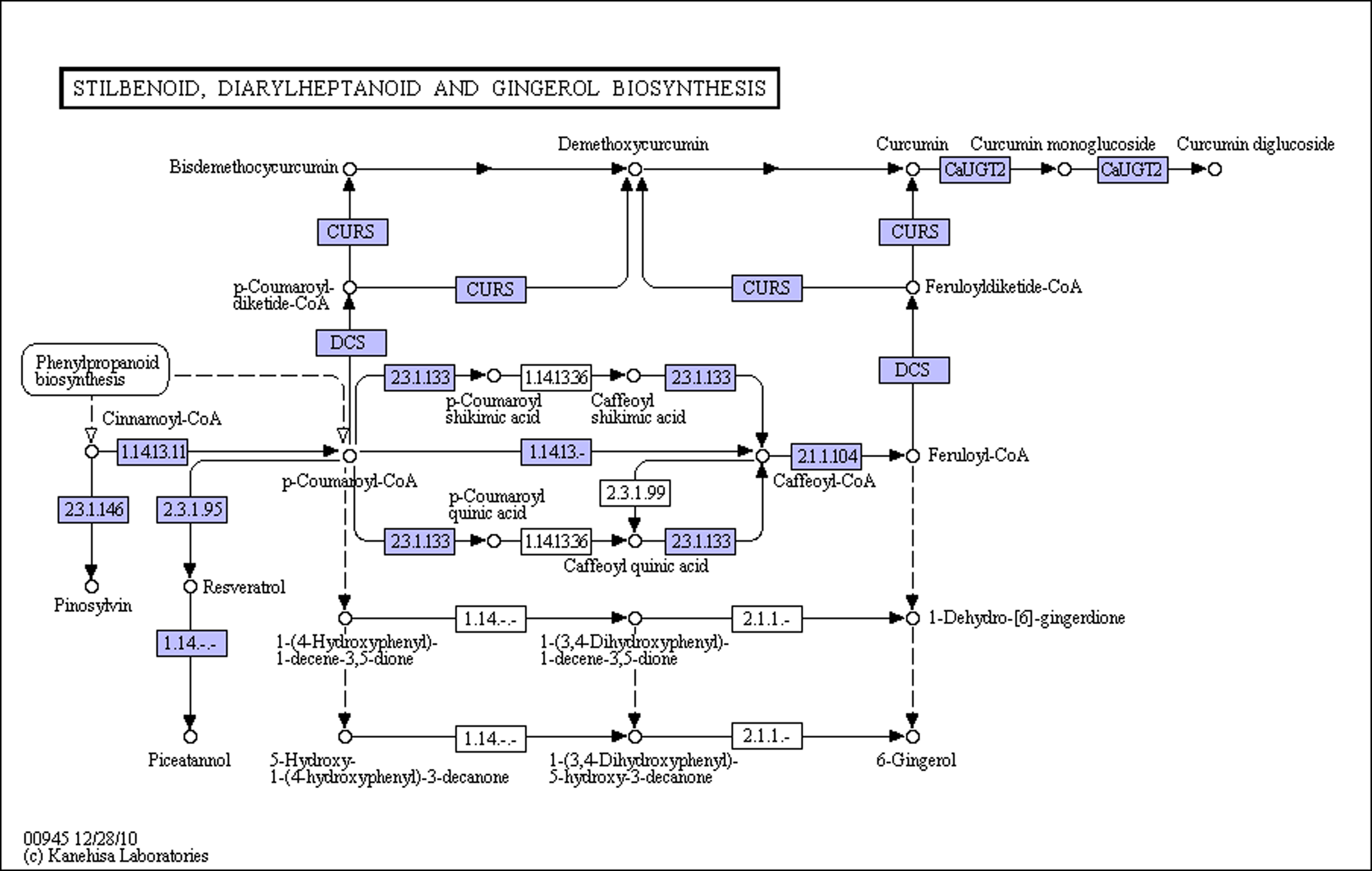

Supplement: Figure S9 — KEGG pathway of bstilbenoid, diarylheptanoid and gingerol biosynthesis. (TIF) [file pone.0097487.s009.tif]

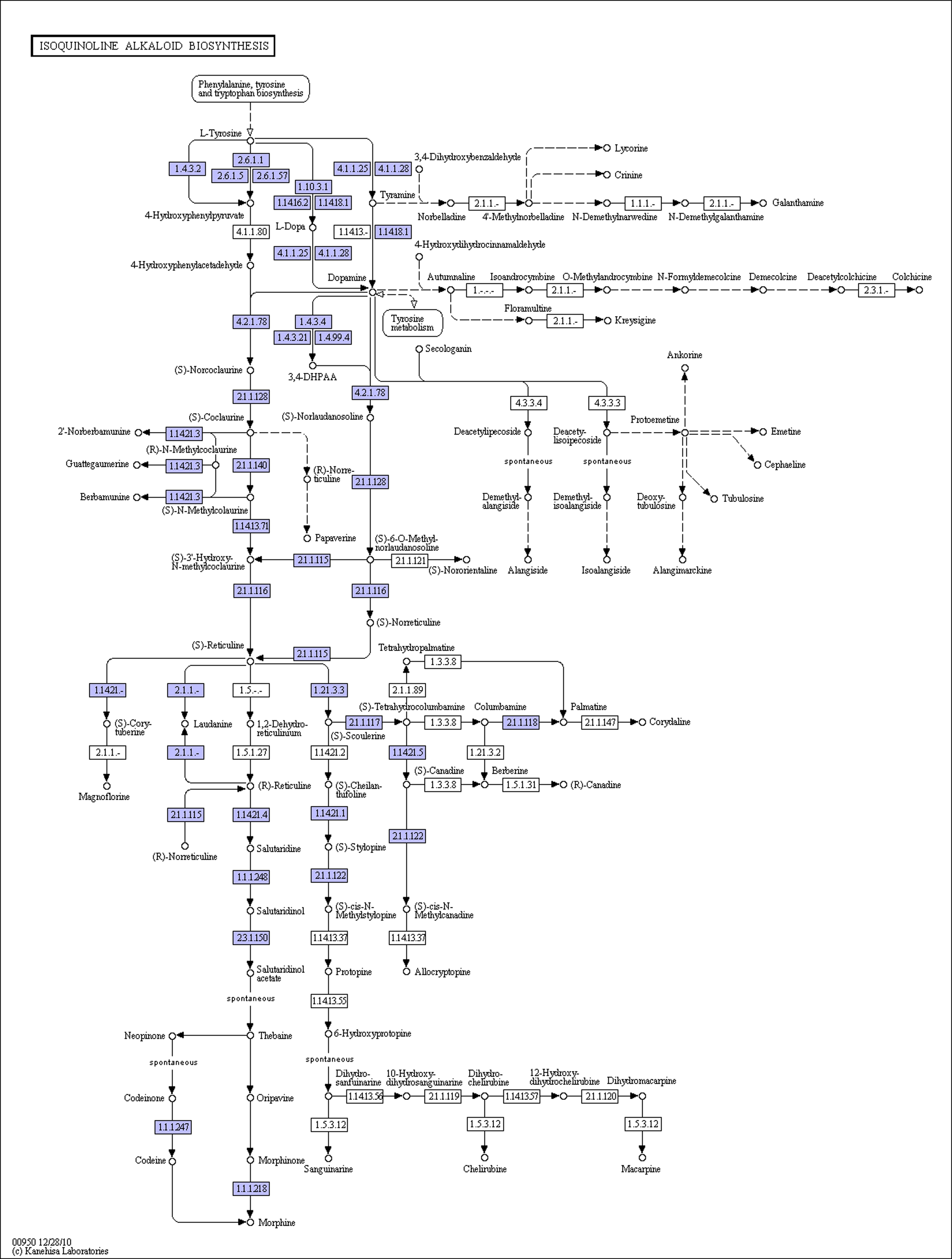

Supplement: Figure S10 — KEGG pathway of isoquinoline alkaloid biosynthesis. (TIF) [file pone.0097487.s010.tif]

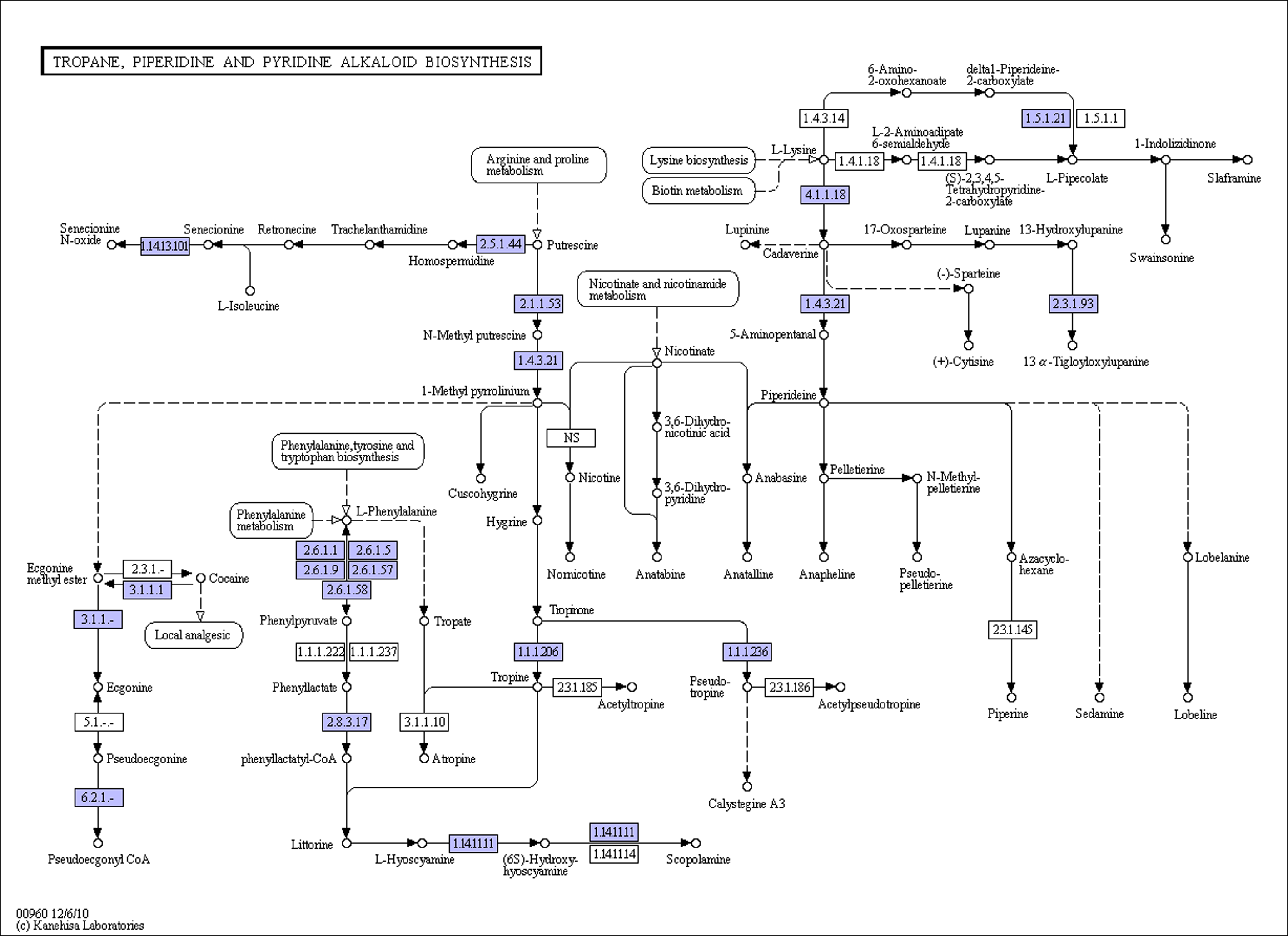

Supplement: Figure S11 — KEGG pathway of tropane, piperidine and pyridine alkaloid biosynthesis. (TIF) [file pone.0097487.s011.tif]

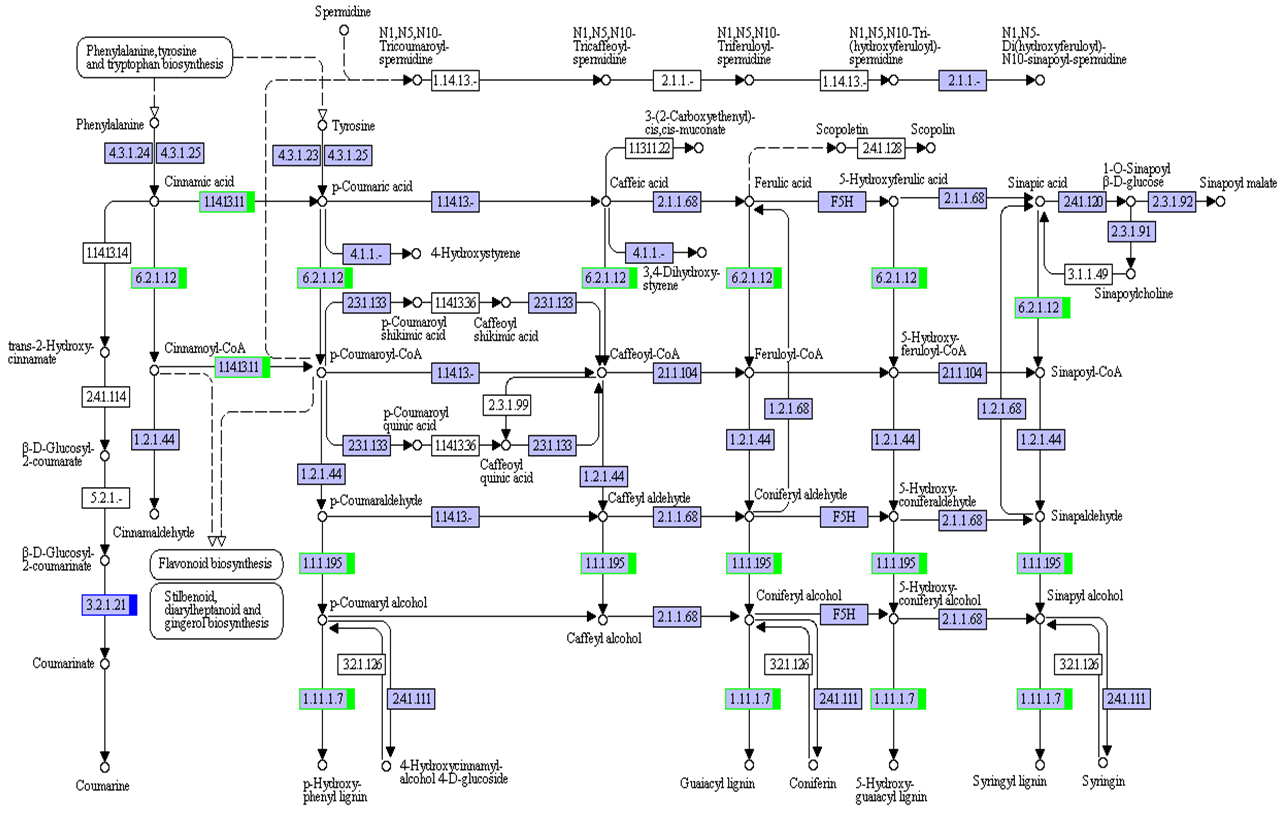

Supplement: Figure S12 — Lignin biosynthesis pathway of DEGs between leaf and root. The gray background was the unigene that was detected in this study. The green background was the unigene that was higher expressed in root than that in leaf. They were listed as follows: [EC:1.14.13.11] trans-cinnamate 4-monooxygenase [EC:6.2.1.12] 4-coumarate–CoA ligase [EC:1.1.1.195] cinnamyl-alcohol dehydrogenase [EC:1.11.1.7] peroxidase. (TIF) [file pone.0097487.s012.tif]

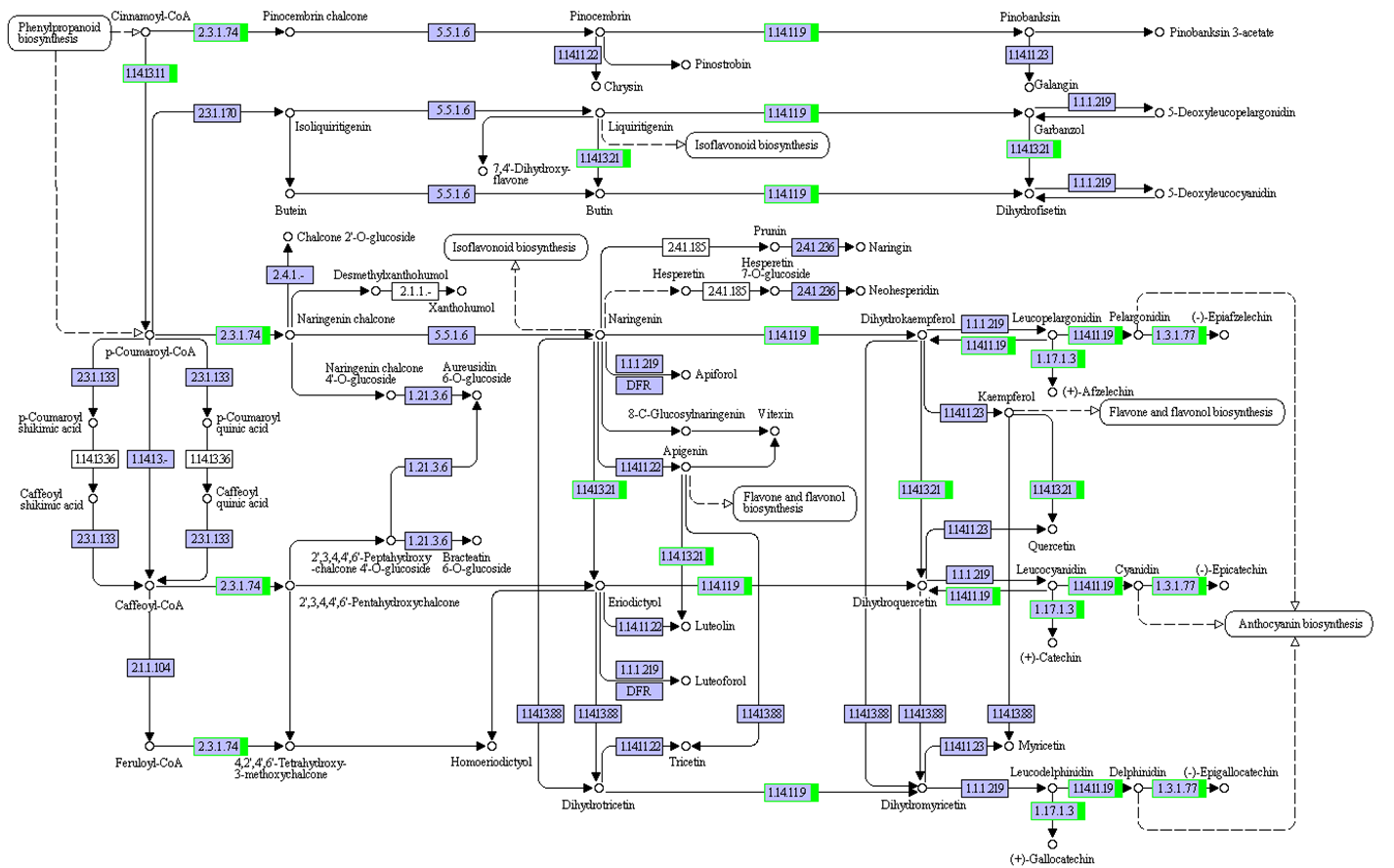

Supplement: Figure S13 — Flavonoid biosynthesis pathway of DEGs between leaf and root. [EC:2.3.1.74] chalcone synthase [EC:1.14.11.9] naringenin 3-dioxygenase [EC:1.14.13.21]flavonoid 3′-monooxygenase [EC:1.14.13.11]trans-cinnamate 4-monooxygenase [EC:1.17.1.3] leucoanthocyanidin reductase [EC:1.14.11.19] leucoanthocyanidin dioxygenase [EC:1.3.1.77] anthocyanidin reductase. (TIF) [file pone.0097487.s013.tif]
