# Supplementary material for: De Novo Assembly of Expressed Transcripts and Global Transcriptomic Analysis from Seedlings of the Paper Mulberry (Broussonetia kazinoki x Broussonetia papyifera)
Source: PLoS One. 2014 May 21;9(5):e97487. doi: 10.1371/journal.pone.0097487 (PMC4029624; doi:10.1371/journal.pone.0097487)
Supplement: Table S1 — Genes chosen for validation. (DOCX) [file pone.0097487.s014.docx]

| ID | Length | Annotation |
| --- | --- | --- |
| T2-26944 | 1115 | Fruit protein |
| T3-23528 | 1299 | 11-beta-hydroxysteroid dehydrogenase 1B |
| T4-18301 | 1917 | Carbonic anhydrase, chloroplastic |
| T4-25084 | 3290 | Ultraviolet-B receptor UVR8 |
| T5-23895 | 2146 | Glutamate--glyoxylate aminotransferase 2 |
| T6-17224 | 1646 | Shikimate O-hydroxycinnamoyltransferase |
| T6-20350 | 1918 | protein phosphatase 2C 25 |
| T6-28644 | 5277 | Zeaxanthin epoxidase, chloroplastic |
| T7-23696 | 1636 | Beta-carotene isomerase D27, chloroplastic |
| T7-23802 | 1805 | Glyceraldehyde-3-phosphate dehydrogenase B, chloroplastic |
| T7-24767 | 1755 | Serine--glyoxylate aminotransferase |
| T7-26228 | 2802 | Cellulose synthase-like protein H1 |

Table S1 Genes chosen for validation
