# Supplementary material for: De Novo Assembly of Expressed Transcripts and Global Transcriptomic Analysis from Seedlings of the Paper Mulberry (Broussonetia kazinoki x Broussonetia papyifera)
Source: PLoS One. 2014 May 21;9(5):e97487. doi: 10.1371/journal.pone.0097487 (PMC4029624; doi:10.1371/journal.pone.0097487)
Supplement: Table S2 — Primers for PCR. (DOCX) [file pone.0097487.s015.docx]

Table S2 Primers for PCR

| Name of primer | Sequence of primer (5′-3′) |
| --- | --- |
| T2-26944-F | TACCCAAATTGACCATTCAAACC |
| T2-26944-R | GGAAGCTGAAGCACCAAAGG |
| T3-23528-F | CTTGGTAGGGTAAGCCATGAACC |
| T3-23528-R | CAGTAAACCACTTTGGACAACTGG |
| T5-18665-F | AATCCCTTCATTGATCTCCACC |
| T5-18665-R | AAGAGCCACAGGCACTTGC |
| T4-25084-F | CTAGCACAATCATAACACCGTTGC |
| T4-25084-R | ACCATTGGAGAGTTACAAAAGCACC |
| T5-23895-F | GAAGAGCAAGGTTAATGACTGATGG |
| T5-23895-R | GGTTGTCCTCAAGTGGAACACC |
| T6-17224-F | GACGGAAGGACTAGGCTACAACC |
| T6-17224-R | GAAATCCCAAGATTAGGGCACC |
| T6-20350-F | CTAAAGAAGGGAACAGAGGATTTGC |
| T6-20350-R | CTTCCATCATCAAACACCATCG |
| T6-28644-F | ATGGATAACCTGCCTTTTTCTGC |
| T6-28644-R | CTGCTTTGGCTTAGCTTAATCTCG |
| T7-23696-F | TCAGCCAATGTCTCAATTTCTAAGG |
| T7-23696-R | ATTCAACAGCAGGAAAACATAATCC |
| T7-23802-F | ATGTTGAGAAGAAGGGCATCTCC |
| T7-23802-R | AGAGGGACATCGCAGACATCC |
| T7-24767-F | TGCTGAATAACATCCCTCTCATCC |
| T7-24767-R | GCAAATTAAGCCTCAAAAGAGTGC |
| T7-26228-F | CTTAACAGCCTTGGCATTGAGC |
| T7-26228-R | CATATTTTCCCTTTCCAAACAACC |
| GAPDH-F | GTTTGATGACCACTGTCCATGC |
| GAPDH-R | GCTGCTAGGAATGATGTTGAAGC |
