# Supplementary material for: De Novo Assembly of Expressed Transcripts and Global Transcriptomic Analysis from Seedlings of the Paper Mulberry (Broussonetia kazinoki x Broussonetia papyifera)
Source: PLoS One. 2014 May 21;9(5):e97487. doi: 10.1371/journal.pone.0097487 (PMC4029624; doi:10.1371/journal.pone.0097487)
Supplement: Table S3 — The sequencing data output and quality assessment on the RNA-seq results of paper mulberry. (DOCX) [file pone.0097487.s016.docx]

Table S3 The sequencing data output and quality assessment on the RNA-seq results of paper mulberry

| Sample | Style | reads1 length | Reads2 length | Base number | Percentage of clean data (%) | GC (%) | Q20 (%) |
| --- | --- | --- | --- | --- | --- | --- | --- |
| Root | raw | 100 | 100 | 1706251159 | 100 | 49.35 | 96.05 |
| Root | clean | 100 | 100 | 1635592137 | 95.86 | 49.22 | 97.77 |
| stem | raw | 100 | 100 | 1927989751 | 100 | 47.96 | 96.35 |
| stem | clean | 100 | 100 | 1856701899 | 96.3 | 47.85 | 97.89 |
| leaf | raw | 100 | 100 | 2059512444 | 100 | 50.1 | 95.92 |
| leaf | clean | 100 | 99 | 1971573613 | 95.73 | 49.91 | 97.65 |
