# Supplementary material for: De Novo Assembly of Expressed Transcripts and Global Transcriptomic Analysis from Seedlings of the Paper Mulberry (Broussonetia kazinoki x Broussonetia papyifera)
Source: PLoS One. 2014 May 21;9(5):e97487. doi: 10.1371/journal.pone.0097487 (PMC4029624; doi:10.1371/journal.pone.0097487)
Supplement: Table S4 — The assembled unigenes results of the RNA-seq results of paper mulberry. (DOCX) [file pone.0097487.s017.docx]

|  | Root | Stem | Leaf | All |
| --- | --- | --- | --- | --- |
| Count | 34213 | 31565 | 27048 | 67443 |
| N50_Length | 1335 | 1349 | 1078 | 1340 |
| Mean_Length | 847.2258 | 842.1836 | 737.1328 | 723.2144 |
| Total_Length | 28986135 | 25005273 | 19795762 | 48775751 |

Table S4 The assembled unigenes results of the RNA-seq results of paper mulberry
