# Supplementary material for: De Novo Assembly of Expressed Transcripts and Global Transcriptomic Analysis from Seedlings of the Paper Mulberry (Broussonetia kazinoki x Broussonetia papyifera)
Source: PLoS One. 2014 May 21;9(5):e97487. doi: 10.1371/journal.pone.0097487 (PMC4029624; doi:10.1371/journal.pone.0097487)
Supplement: Table S13 — GO terms related to the stress response. (DOCX) [file pone.0097487.s026.docx]

| number | GO term |
| --- | --- |
| 368 | activation of immune response |
| 6526 | cellular response to stimulus |
| 1043 | negative regulation of response to stimulus |
| 2687 | regulation of response to stimulus |
| 722 | positive regulation of response to stimulus |
| 9511 | response to stress |
| 3436 | immune response |
| 3716 | response to external stimulus |
| 5059 | response to biotic stimulus |
| 6171 | response to abiotic stimulus |
| 4557 | response to endogenous stimulus |
| 8481 | response to chemical stimulus |
| 770 | detection of stimulus |
| 6526 | cellular response to stimulus |
| 794 | interspecies interaction between organisms |
| 4521 | response to other organism |

Table S13 GO terms related to the stress response
